# Nodulation number tempers the relative importance of stochastic processes in the assembly of soybean root-associated communities

**DOI:** 10.1038/s43705-023-00296-8

**Published:** 2023-08-28

**Authors:** Lei Wang, Yan Jiao, Yingdong Bi, Yanli Hu, Yan Jiang, Shaodong Wang, Sui Wang

**Affiliations:** 1 Key Laboratory of Soybean Biology in Chinese Ministry of Education, Northeast Agricultural University, 150030 Harbin, PR China; 2 School of Resources and Environment, Northeast Agricultural University, 150030 Harbin, PR China; 3 Institute of Crop Cultivation and Tillage, Heilongjiang Academy of Agricultural Sciences, 150028 Harbin, PR China

**Keywords:** Soil microbiology, Microbial ecology

## Abstract

Identifying the ecological forces that structure root-associated microbial communities is an essential step toward more sustainable agriculture. Legumes are widely utilized as model plants to study selective forces and their functioning in plant-microbial interactions owing to their ability to establish mutualism with rhizobia. Root nodules act as symbiotic organs to optimize the cost-benefit balance in this mutualistic relationship by modulating the number of nodules. However, it is not known whether the number of nodules is related to the structure of root-associated bacterial communities. Here, the root-associated bacterial communities of soybean grown in native soil by means of soybean cultivars with super- or normal nodulation were investigated across four developmental stages. We compared ecological processes between communities and found decreased relative importance of neutral processes for super-nodulating soybean, although the overall structures resembled those of normal-nodulating soybean. We identified the generalist core bacterial populations in each root-associated compartment, that are shared across root-associated niches, and persist through developmental stages. Within core bacterial species, the relative abundances of bacterial species in the rhizosphere microbiome were linked to host-plant functional traits and can be used to predict these traits from microbes using machine learning algorithms. These findings broaden the comprehensive understanding of the ecological forces and associations of microbiotas in various root-associated compartments and provide novel insights to integrate beneficial plant microbiomes into agricultural production to enhance plant performance.

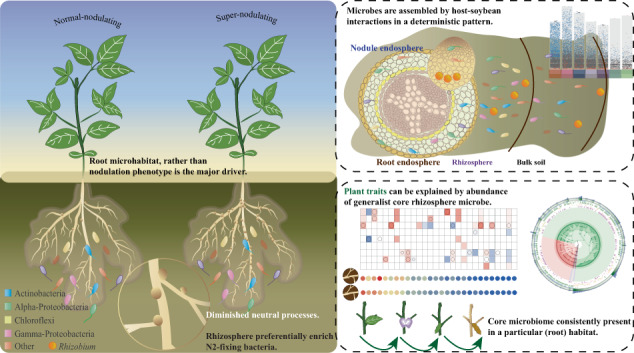

## Introduction

The plant microbiome is an integral part of the plant “holobiont”, which has coevolved with its host and affects plant traits in profound ways [1]. Nevertheless, there is a lack of fundamental understanding of the ecological forces that structure these communities, and need a comprehensive identifying of the patterns and principles that shape the microbiome. As generally accepted, both deterministic and stochastic processes are thought to shape microbial community composition [2]. Plant-associated microbiomes begin to form shortly after sowing and develop with plant growth through four eco-evolutionary processes: ecological drift, speciation/diversification, dispersal, and selection [3, 4]. Speciation and dispersal can be both stochastic and deterministic, while ecological drift and selection are clearly stochastic and deterministic, respectively. The relative contributions of stochastic and deterministic processes and how they cooperate to regulate community establishment remain critical questions.

Of note, for many legumes, root nodule symbiosis defines a specific plant-microbe interaction where rhizobial colonization initiates in root hairs via transcellular infection threads [5, 6]. This complicates the assembly pattern of the community [7–10]. Legumes, unlike most land plants, can develop root nodules to cope with the presence of their bacterial partners. The formation of these de novo lateral root organs houses the microsymbionts inside their host cells and provides a means to control the mutualism between microbes and legumes [11, 12]. During nodule development, cortical cells that divide confined endosymbiotic bacteria in root nodules, with the bacteria-induced signal cascade and the host-controlled autoregulation of nodulation (AON), accurately control the number of nodules and optimize the mutualistic relationship’s cost-benefit balance [13–15]. The disorder in autoregulation of nodulation often leads to an abnormal number of nodules. Perturbations in mutualistic relationships are not limited to host legumes and may also affect the known composition of the root-associated bacterial communities and drive their distribution, which may extend influence to other crops intercropped or rotated with legumes [7, 8]. This information is important for comprehending the symbiotic evolution of legumes and engineering symbiotic biological nitrogen fixation (BNF) in nonlegume plants to incorporate these specialized binary interactions into the context of an ecological community.

Derived from the field of ecology, generalists refer to taxonomic groups capable of thriving in diverse environments or gradients [16, 17]. Within this group, certain taxa form part of the core microbiome, persistently present across multiple assemblages within a habitat, such as root [16, 18, 19]. Hence, despite the considerable variation in the definition of core microbiome across studies encompassing different species and scales, it primarily refers to persistent and occasionally highly abundant microbial taxa within a microbial community that are also considered as stable consortia. Still, a stable presence does not mean that the abundance of these core microbes is constant. Although research during the past few years has generally accepted the potential importance of the generalist core microbiome members for developing solutions for sustainable crop production systems, the differences between samples and its capacity to predict plant phenotypes remains elusive.

## Materials and methods

### Soil characterization and plant material

Test soils were collected from the surface layer (0–15 cm) of a soybean field located in Harbin, Heilongjiang Province (45°44′N, 126°43′E). HZ 9009 is a super-nodulating soybean cultivar (hereafter SNS) with obviously more nodules than common cultivars. According to the classification for maturity of SNS, Heihe 43 (normal-nodulating soybean, hereafter NNS) was selected as a candidate control variety, which is one of the most widely cultivated varieties in the Fourth Accumulative Temperature Belt in Heilongjiang Province. The genetic distance was calculated using the whole-genome *k*-mer comparison method based on re-sequencing data (Fig. S1) [20]. Supplementary Material A and B provide detailed characters and potential molecular mechanisms underlying the variation in nodule number between the two cultivars. See Supplementary Material C for relevant descriptions of the topsoil property, soybean cultivation, sampling and plant trait measurement.

### DNA extraction, PCR amplification and sequencing

In total, extractions were performed from 0.35 g of homogenized bulk soil samples, rhizosphere samples, root samples, and nodule samples using the E.Z.N.A.®Soil DNA Kit (Omega Bio-Tek, Norcross, GA, U.S.) following the manufacturer’s instructions. Quantification was performed using an agarose gel and a Nanodrop spectrophotometer (Thermo Scientific, DA, USA). To avoid amplifying mitochondrial DNA and nontarget DNA in plant endosphere, hypervariable V5–V7 regions of the 16S rRNA gene were amplified with barcoded PCR primers 799 F [21] and 1193 R [22]. The cycling conditions consisted of 2 min at 98 °C, 27 cycles of 15 s at 98 °C, 30 s at 55 °C, 30 s at 72 °C, and 5 min at 72 °C. The reactions were run in 25 μL mixtures containing 5 μL of 5 × reaction buffer, 5 μL of 5 × GC buffer, 2 μL of 2.5 mM dNTPs, 1 μL of 10 μM each primer, 2 μL of DNA Template, 8.75 μL of ddH_2_O, and 0.25 μL of Q5 DNA Polymerase. Finally, Paired-end sequencing (2 × 250 bp) was conducted on an Illumina NovaSeq 6000 platform for short-read sequencing by Frasergen Bioinformatics Co., Ltd. (Wuhan, China).

### 16 S rRNA gene sequencing data collection and processing

Demultiplexed raw sequence data were processed using QIIME2 (version 2019.04). DADA2 pipeline was used to denoise the initial raw reads into amplicon sequence variants (ASVs) [23], followed by filtering and chimera removal with default settings. Taxonomy assignments were performed using a naïve Bayes classifier trained by SILVA 16 S rRNA database in QIIME2, excluding unclassified ASVs below the domain *Bacteria* level from further analyses. Sequencing depth was normalized based on the 95th percentile of the lowest sampling depth, and a phylogenetic tree was built using the fragment insertion function in QIIME2 with the original SILVA tree as a starting tree. Finally, a single phyloseq object was created using the phyloseq R package (R Version 4.1.0), consisting of an aggregated mapping file, the phylogenetic tree, the taxonomy table, and the ASV table. See Supplementary Material D for relevant descriptions of basic survey of 16S datasets.

### Diversity and assembly pattern analyses

Both α-diversity (Chao1 index) and β-diversity (Bray‒Curtis dissimilarity and Jaccard similarity index) analyses were performed using the R package “phyloseq”. Nonparametric statistical tests (i.e., Kruskal–Wallis tests) were used to test for group differences. The major drivers of the α-diversity index were identified with a variance partitioning analysis (VPA) using the R package “rdacca.hp” [24]. The variable strength was quantified using repeated-measures analysis of variance (ANOVA). The relative contribution of different factors to the bacterial community dissimilarity was determined with permutational multivariate analysis of variance (PERMANOVA, permutation = 999) using the R package “vegan” [25].

Two methods were employed to infer the assembly process of root-associated microbiome. Firstly, beta nearest taxon index (βNTI) was calculated to evaluate the relative importance of stochastic and deterministic processes in bacterial community assembly using a null model with 999 randomizations [26]. This metric compares observed β-mean nearest taxon distance (βMNTD) to the null expectation estimated by breaking dendrogram association [27]. The standard deviation was normalized using R package “picante”. Secondly, a neutral community model (NCM) was used to predict the correlation between detection frequency of ASVs and relative abundance in metacommunity [28], aiming at determining potential significance of neutral processes. Based on neutral theory, NCM assumes that abundant species in metacommunity would be randomly distributed by individuals while rare species are susceptible to being lost in various local communities due to ecological drift. Parameter values *R*^2^ and m quantified fit to model and its migration rate respectively. The model was implemented in R using “stats4” package and fit using “minpack.lm” package [29].

### Association analyses of microbes inhabiting continuous rhizo-compartments

To investigate the relationship between the root endosphere and the soil, a Bayesian bacterial source-tracking model was implemented to estimate the potential sources of microbiota inhabiting each root-associated compartment [30]. Differentially abundant ASVs were detected using DESeq2’s negative binomial generalized linear model approach. ASVs were considered enriched if they had a log_2_-fold change >2 and an FDR-adjusted *p* value (*q* value) <0.05 [31].

### Definition of the generalist core microbe

Generalists and specialists were classified based on previous studies [18, 19, 32]. The Levins indices were used to estimate population niche width by analyzing the frequencies of bacterial taxa across different plant developmental stages within each microhabitat [33]. The occurrences of ASVs were derived by simulating 1000 permutations using the R package “EcolUtils” [34]. ASVs with higher niche width values are considered generalists as they use a broader range of habitats. Specialists are those whose observed occurrence falls below the lower 95% confidence interval while generalists exceed the upper 95% confidence interval. Procrustes rotations were performed to test pairwise concordance between generalists and the total community. The taxonomic cladogram of generalist species was visualized using GraPhlAn [35], and functional annotation was predicted by FAPROTAX database [36]. Generalist core microbes refer to those present in samples across all developmental stages for each root-associated niche and associated bulk soil. Differences in relative abundance of these microbes were visualized using a heat tree created with R package ‘metacoder’ [37].

### Prediction of soybean phenotype by root-associated core microbiome

Plant trait data are provided in Supplementary Material Table S1. Correlations between the relative abundance of generalist species inhabiting each root-associated compartment and host-plant traits were determined by Spearman nonparametric analyses. Machine learning algorithms were used to investigate whether generalist species explain plant functional traits, with the abundance of each microbiota acting as a predictor of plant functional traits. The predictive model was created by performing the random forest analysis using the R package ‘randomForest’. This approach was chosen due to its sensitivity for predicting environmental bacterial datasets [38]. The importance of each generalist microbe was evaluated by the decrease in prediction accuracy. The significance of each predictor for the response variables was evaluated using the package ‘rfPermute’.

## Results

### Diversity and composition of the root-associated microbiome in different nodulation phenotypes

Thes result of whole-genome *k*-mer comparison among SNS, NNS, Zhonghuang 13 and Williams 82 are depicted in Fig. S1, revealing minimal genetic variation between the two soybean cultivars. (Supplementary Material A, B).

**Fig. 1:**
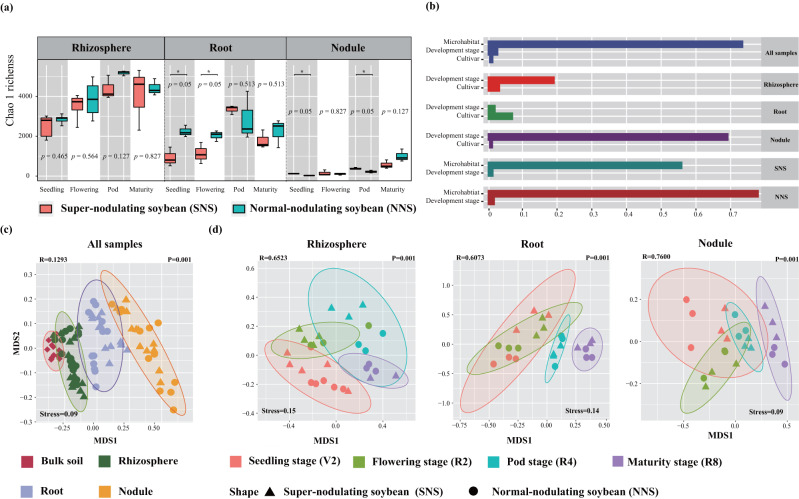
Temporal dynamics of diversity and distribution patterns of root-associated bacterial communities. **a** Boxplot of α-diversity of bacterial communities in root-associated microhabitats and bulk soils across four plant developmental stages. *P < 0.05, Kruskal–Wallis, Dunn posttest. **b** Effects of multiple factors on bacterial α-diversity. **c** NMDS plot shows the Jaccard dissimilarity of bacterial community compositions along the soil-root continuum at the ASV level. **d** NMDS ordinations based on Jaccard distance of bacterial communities in each microhabitat.

A three-way ANOVA revealed that α-diversity of the microbial community varied by developmental stage, soybean cultivar, and microhabitat (Table S3, *P* < 0.05). NNS soybean yielded higher bacterial Chao 1 richness than SNS soybean, especially in the plant endospheric niches at the seedling stage (Fig. 1a). The variance partitioning analysis indicated that plant developmental stage had a greater influence on nodule microhabitats than on root and rhizosphere microhabitats, and microhabitat variety was the dominant factor that explained 73.64% of the bacterial Chao 1 richness variation (Fig. 1b). Furthermore, it is important to note that only in the root microhabitat did the cultivar factor contribute more than the plant developmental stage to the dynamics of Chao 1 richness.

PERMANOVA and NMDS ordination indicated that the bacterial communities differed significantly between microhabitats, which suggested a clear spatial compartmentalization of the bacterial microbiota (Jaccard dissimilarity, *R*^2^ = 0.12926, *P* = 0.001; Bray‒Curtis dissimilarity, *R*^2^ = 0.42986, *P* = 0.001; PERMANOVA) (Fig. 1c, Table S4). The microbiome samples from four plant developmental stages separated along the principal coordinate, in agreement with PERMANOVA statistic (Jaccard dissimilarity, *R*^2^ = 0.07521, *P* = 0.001; Bray‒Curtis dissimilarity, *R*^2^ = 0.09224, *P* = 0.007; PERMANOVA) (Fig. 1d, Table S4). The PERMANOVA analysis, using a Jaccard dissimilarity measure, indicates that the nodulation phenotype had a minimal overall effect on the root-associated microbiota (*R*^2^ = 0.01584, *P* = 0.048). However, when employing a Bray‒Curtis dissimilarity measure in the PERMANOVA analysis, the differences between different cultivars cannot be considered statistically significant (*R*^2^ = 0.16889, *P* = 0.078) (Table S4). This discrepancy is likely due to differences between the Jaccard and Bray distance metric: nodule phenotype has a greater impact on rare taxa frequency than abundant taxa, resulting in a larger effect size for cultivar with Jaccard. Besides PERMANOVA, we also used the Bray-Curtis dissimilarity index to compare beta-diversity temporal dynamics among different cultivar groups (Fig. S7). Plant endophytes in the NNS group exhibited higher beta-diversity compared to those in the SNS group, indicating greater dispersion.

The weighted beta-NTI indicated that deterministic processes played a more significant role in the assembly of the root-associated microbiome (|βNTI | ≥ 2), with the nodule having the lowest stochasticity ratio (Fig. S8a). The observation matched NCM results, deterministic processes strongly affected soybean bacterial communities, particularly in nodules, consistently across cultivars and developmental stages (Fig. S8b). However, the relative importance of neutral processes was lower in the bacterial community inhabiting soybean roots with a supernodulated phenotype.

### The variation in microbiomes resulting from differences in nodulation number throughout plant development stage

Canonical analysis of principal coordinates (CAP) was conducted using Jaccard metrics to determine the variance attributable to nodule number (Supplementary Material C). The CAP results indicated that nodule number did not significantly account for beta-diversity of nodule endophytes at both ASV and genus resolution, while it had a significant effect on root endophytes (Table S5). In addition, rhizosphere microbial communities were also influenced by nodulation number, albeit only at ASV resolution. This finding was supported by the Mantel test (Table S6).

To explain which taxa accounted for the phenotypic effects in each microhabitat, we performed differential genus abundance analyses between the cultivars. In total, none of identified genus were significantly affected by the cultivars. Although *Bradyrhizobium* display relatively higher in SNS at each microhabitat but cannot be considered as statistically significant (Fig. S9). Still, we noted that *Bradyrhizobium* played a significant role in the differences among root-associated compartments and throughout various stages of plant development (Fig. S10a). We subsequently evaluated the temporal variation in *Bradyrhizobium* prevalence within the nodule microhabitat during nodule senescence. In general, *Bradyrhizobium* significantly decreased during the maturity stage, whereas *Chryseobacterium* and other genera exhibited a notable increase, suggesting a transition from nitrogen fixation to saprophytic taxa (Fig. S10b). This variability may be attributed to the plant’s nutrient acquisition strategies. Principal Component Analysis (PCA) was next performed on the ASV members of *Bradyrhizobium*, revealing a clear separation between SNS and NNS at the maturity stage (Fig. S10c).

### Linkages of the microbiome inhabiting different root-associated microhabitats

**Fig. 2:**
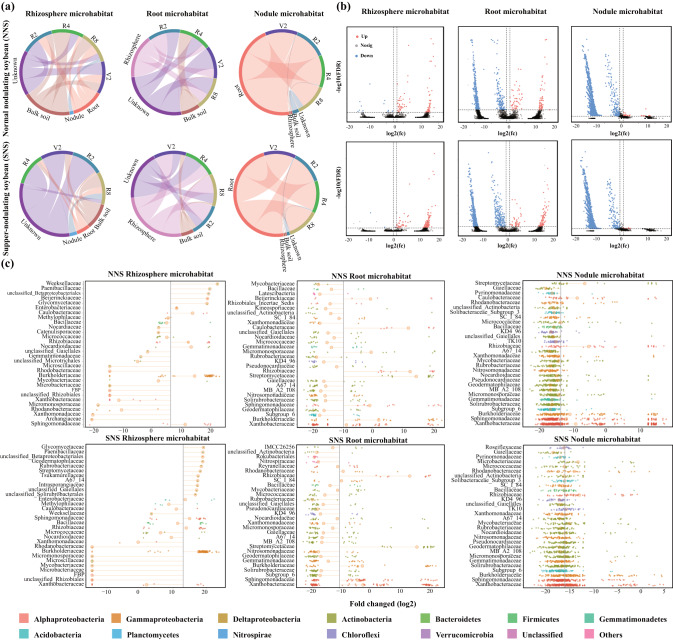
The microbiota association within distinct root-associated microhabitats. **a** Source tracking of microbe across root-associated microhabitats. **b** Root-associated microhabitats are delepted and enriched for certain ASVs. The abundance fold change compared to bulk soil is represented by the position along the *y* axis, while each point represents an individual ASV. **c** Top 30 depleted or enriched ASVs between each microhabitat compared with bulk soil.

The predicted sources of bacterial microbiota for each microhabitat varied among individual subjects, but the majority of the bacterial microbiotas within each nodule microhabitat were attributable to the root habitat (81.1% on average), and root microhabitats appeared to both receive microbiota from rhizosphere (43.0% on average) and contribute to the rhizosphere microbiotas (17.4% on average) (Fig. 2a). To identify significantly enriched ASVs that are associated with different microhabitats, the enrichment and depletion of specific ASVs in each root-associated microhabitat compared with the bulk soil microhabitat was determined using DESeq2 analysis (Fig. 2b). The rhizosphere microhabitat compartment was the most similar to the bulk soil compartment; however, an enrichment effect in the rhizosphere microhabitat is represented by a highly significantly enriched/depleted ratio of ASVs. In contrast, while the root endosphere enriches for many ASVs (111 for NNS and 126 for SNS, mainly *Streptomycetaceae* and *Rhizobiaceae*), it also depletes a larger proportion of ASVs (541 for NNS and 826 for SNS, mainly *Xanthobacteraceae and Subgroup_6*). The nodule microhabitat represents an exclusive niche with a minimal ratio of enriched ASVs compared to other niches (NNS: 241 vs. 3955 and SNS: 13 vs. 3972), mainly enriched with *Burkholderiaceae, Enterobacteriaceae, Rhodanobacteraceae, and Xanthobacteraceae* (Fig. 2c; Fig. S11). While the overwhelming majority of microbiotas depleted in the nodule microhabitat were also depleted in the root microhabitat, there were minimal overlapping enriched ASVs in the two compartments (Fig. S12), each of which appears to reflect a different underlying recruitment mechanism. Moreover, despite an overall similarity in the recruitment patterns from each cultivar, distinct differences were observed in nodule microhabitat, where the NNS nodule microhabitat showed a higher attraction of diverse and abundant taxa compared to SNS. This observation may be relevant for the unique nutrient availability in nodule microhabitat, and complex recognition mechanisms of host plants.

### Generalist core microbiota in soybean roots

**Fig. 3:**
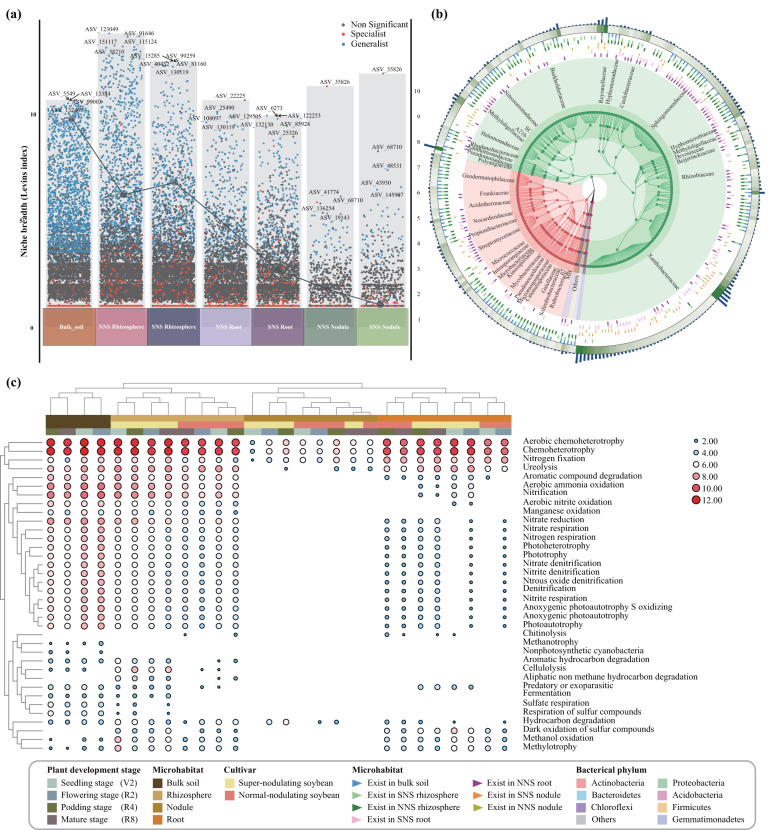
Abundance, phylogeny, and potential functions of generalist microbes in distinct root-associated compartments. **a** Niche width of the overall bacterial communities based on Levins index. Specialists are defined as those with the smallest niche width, and generalists as those with the largest niche width. Line graph represents the generalist/specialist ratios. **b** Phylogenetic tree and taxonomic distribution of generalist microbes. **c** Heat map of the relative abundance of functional gene families inferred by FAPROTAX function prediction.

Generalists and specialists were found in all of these diverse microhabitats and their distribution was clearly affected by root microhabitats. The largest prevalence values of generalists are shown over rhizosphere compartments (7.9% for NNS and 6.5% for SNS), while nodule habitats have the largest specialist prevalence values (Fig. 3a). In addition, the averaged community niche breadth (Levin’s index) of the rhizosphere microbiotas was significantly highest among all root-associated microbiotas, suggesting that it may be more metabolically flexible at the community level.

We next investigated the linkages of generalist microbiomes with the overall bacterial communities. As illustrated in Fig. S13, with the exception of the nodule microhabitat groups, there was a strong correlation between the generalist microbiota and the overall bacterial communities, as indicated by the high similarity in beta-diversity. In addition, clear differences were observed in the taxonomic structure and putative function of generalist microbiotas in different microhabitats (Fig. 3b, c).

**Fig. 4:**
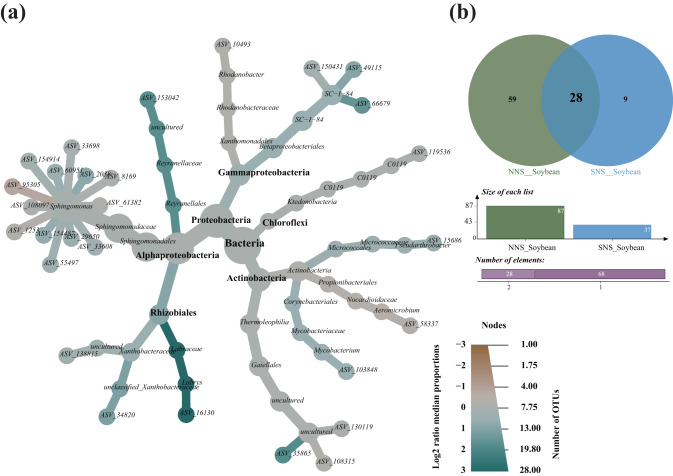
Taxonomic distribution of core generalist microbes. **a** Heat Tree of composition of the 28 shared core generalist microbes with comparisons between the two cultivars. The node size corresponds to the number of ASVs identified within a given taxonomic group, while Log2 changes were determined through Wilcoxon rank-sum testing. The color assigned to each taxon reflects differential abundance between two cultivars, with colors determined by the log2 ratio of median proportions of reads observed in each feeding type. **b** The Venn diagram illustrates the quantity of common generalist microbes shared between both cultivars. *NNS* normal-nodulating soybean, *SNS* super-nodulating soybean.

Only 87 generalist ASVs for NNS soybean and 37 generalist ASVs for SNS soybean were found to be present in all the developmental stages and overlapped among the bulk soil, rhizosphere and root endosphere microhabitats, therefore falling into the core generalist microbe definition (Fig. S14). The majority of the ASVs belonged to the Alpha-, Gamma-Proteobacteria, Actinobacteria and Chloroflexi (Fig. 4). At the genus level of taxonomic resolution, the identified ASVs were affiliated with *Sphingomonas*, *SC-I-84*, *uncultured_o__Gaiellales*, *uncultured_f__Xanthobacteraceae*, *Aeromicrobium*, *Rhodanobacter*, *C0119*, *uncultured_f__Reyranellaceae*, and *Pseudarthrobacter*. However, the cluster analyses reveal significant disparities in the core microbiota distribution between the two cultivars (Fig. S15).

### Correlation analysis of parameters

**Fig. 5:**
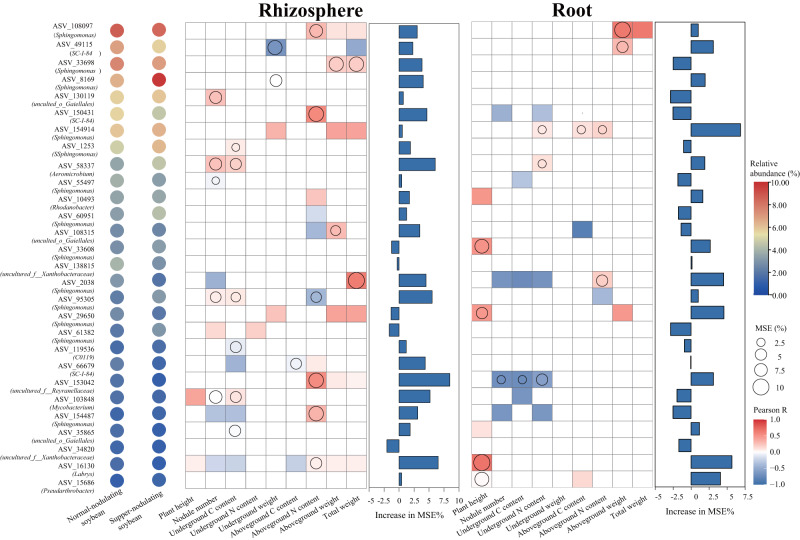
Association between plant phenotypes and relative abundance of generalist core microbes inhabiting rhizosphere and root microhabitats inferred from Spearman analysis and the random forest models. Bubble plot illustrates the distribution of relative abundance of core microbes in each soybean cultivar. the bar diagram displays the relative importance of these core microbes. The relative important of core microbes is shown in bar diagram. Correlation matrix between plant traits and core microbe abundance are shown, with a color gradient denoting Pearson’s correlation coefficient. Only the significant (*P* < 0.05) and strong correlations (correlation > 0.5 or <−0.5) were visualized. Colors represent Spearman correlations. Circle size represents the variable importance of the generalist microbe determined by the random forest model.

To further understand the correlation between core microbes and plant functional traits, Spearman analyses were conducted to capture associations in time series data, and important predictors were identified using random forest analysis. A significantly positive correlation was found between the relative abundance of the major core microbes in rhizosphere microhabitats and one or numerous plant functional traits, including aboveground N content, underground C content, nodule number and plant biomass (Fig. 5). For instance, the relative abundance of several ASVs belonging to the genus *Sphingomonas* were significantly related to plant biomass, underground C content and aboveground N content, and the relative abundance of ASVs of the genus *uncultured_o__Gaiellales* was highly correlated with soybean nodulation. In comparison, in the root endosphere microhabitat, there was a general lack of positive association between core microbes abundance and plant characteristics, with some exceptions. For example, only the relative abundance of ASVs belonging to the genera *Sphingomonas, SC_I_84* and *Labrys* were positively related to plant traits. Similarly, random forest modeling analysis showed that generalist core microbes inhabiting the rhizosphere microhabitat are a stronger predictor of plant functional traits than those in the root endosphere microhabitat.

## Discussion

### Microhabitat filtering, rather than nodulation phenotype, is the major driver shaping root-associated microbial communities

The assembly of the root-associated microbiome is influenced by multiple drivers, such as the environment‒microbe interactions and host signature [39, 40]. In our study, under the greenhouse conditions, the root-associated microhabitat described the largest source of variation in bacterial structures with strong support for microbiome clustering (Fig. 1c). Consistent with previous studies on various plant species, different phyla were found to be distributed within root-associated microhabitats due to niche adaptations [41, 42]. Plant development described the second source of variation in the bacterial communities sampled (Fig. 1d). In accordance with our data, the root-associated microbiomes varied significantly over time, both in terms of α- and β-diversity. These findings further strengthen the idea that the plant host provides a strong selection effect, enabling the establishment of distinct bacterial communities in the different plant development stages [43]. It is noteworthy that in the case of the greenhouse experiment under the same soil conditions, we observed that only in the root endosphere did cultivar factors contribute more than plant developmental stage factors to the bacterial community dynamics (Fig. 1b). These results are in contrast to those found in the long-term coevolution of plant–microorganism studies in which the effect of plant genotype is thought to be the largest source of variation [44]. Recent works on modern agricultural systems and domesticated crops have also revealed that the modulating role of host-plant subspecies/genotypes/cultivars in rhizosphere microbiome assembly is thought to be quite small compared to that of natural systems with a long history of coevolution [19, 45, 46]. However, of note, it is probable that our study underestimates the proportion of variation explained by cultivars. Our 16S survey captures genus-level variation using a highly conserved ribosomal marker gene, but it is likely that there exists finer-scale taxonomic variation. In addition, this study examined only two soybean cultivars with different nodulation phenotypes; thus, our findings cannot account for any variants not represented in these two cultivars.

To identify the effect of nodule numbers on bacterial community assembly, we examined the multifaceted ecological forces in super- and normal-nodulated soybean. The overall structures of the bacterial communities were similar for the two nodulation phenotypes, although we anticipate that nodule numbers would affect the assembly patterns of microbes. Within each nodulation phenotype, the NCM for numerous taxa was found to deviate from neutral predictions, which are ecologically important [29]. However, the neutral processes become relatively diminished for the SNS community, suggesting that hypernodulation may alter the ecological mechanisms of root-associated bacterial community assembly [10, 12, 14]. It is likely that soybean plants lacking autoregulation of nodulation exhibit hypernodulation, and thereby increasing the deterministic components of community assembly relative to the stochastic ones. With this finding in mind, we sought to focus further on the community assembly rules of each root-associated compartment, considering that root-associated microbiome is shaped by the specific microhabitats.

### The assembly of bacterial microbiotas associated with roots was mainly dominated by deterministic processes

Root-associated microbial diversity patterns have been well-documented across different microhabitats [42, 46]. From an initial focus on the description of patterns, interest has recently turned to the role of underlying processes that regulate the assembly of bacterial communities. The continuum hypothesis states that both stochastic and deterministic processes contribute to the assembly of ecological communities [47, 48]. There is little doubt that root-associated microbiota assembly is in reality the result of multiscale processes, and only the relative importance of each is controversial. In our study, depletion and enrichment of certain microbiotas across the microhabitats suggests that the bacterial colonization of soybean roots is not a passive process and that roots have the ability to select for certain members of bacterial consortia (Fig. 2). Similar to studies conducted in *Arabidopsis* [49] and rice [42], our findings indicate a decrease in the relative abundance of Acidobacteria within the root endosphere compared to the surrounding soil. Conversely, we observed an increase in the relative abundance of Proteobacteria from soil to endosphere, suggesting that certain microbial members are better suited for colonizing roots (Fig. S7b). In addition, the results of our study provide quantitative evidence that deterministic processes appear to play a more significant role in shaping microbial community assembly compared to stochastic processes, regardless of root compartment (Fig. S8).

The importance of deterministic processes for shaping rhizosphere microbes has been documented in previous studies. This is noteworthy, as the rhizosphere landscape is strongly influenced by root rhizodeposition from host plant metabolism, and its dynamics can have profound impacts on plant fitness [50, 51]. The root-associated microbiotas have strong chemotaxis activities toward plant signaling molecules. These signaling molecules are established along with the development of plant organs, such as roots and provide a spatially heterogeneous source of nutrients, setting the stage for a shift in selective pressure on the microbes that contributes to the increased significance of selection in the community assembly process [52]. Our findings support previous research indicating that the microbial communities inhabiting the root endosphere exhibit greater niche specialization compared to those in the rhizosphere, suggesting an evolutionary adaptation to overcome plant innate immunity and establish a successful colonization within the endosphere [53]. The mechanisms involved in these putative selections are still being elucidated, but genetic studies to date show that the host genes related to root hair structure, cell walls and disease resistance may contribute [54]. The lowest ratio of stochasticity in the nodule sample suggests that deterministic processes are relatively more important in the nodule endosphere (Fig. S8a). This observation was consistent with the results of the NCM analysis, which showed that the community assembly clearly deviated from the neutral hypothesis in the nodule compartment (Fig. S8b). Nodules represent a truly distinct microhabitat from the adjacent root endosphere by being [1] dominated by monotonous bacterial taxa (i.e., rhizobia), [2] rich in both nitrogen and carbon, and [3] low in oxygen, which is necessary for nitrogen fixation [12, 15]. As such, unlike the taxonomically diverse rhizosphere- and root endosphere-dwelling microbiotas that establish complex networks of microbe‒plant and microbe‒microbe associations, nodule microhabitats define highly specific binary microbe‒plant interactions exhibit stronger selective pressures.

### Soybean phenotypic traits correlated with the generalist core microbes inhabiting rhizosphere

The term core microbiome was aptly coined in recent years to describe the taxa that are consistently present in a particular habitat [18, 55]. These core microbes can be considered generalist species that are present in diverse environmental conditions including individual hosts and through time [16]. Here, a set of such core microbes consisting of generalist species sharing diverse root-associated compartments was probed by defining their niche widths. These core generalist species were persistent through plant developmental stages. Furthermore, at the genus-level annotation, majority of the identified core microbes (e.g., *Sphingomonas*, *Aeromicrobium*, *Pseudarthrobacter, and Rhodanobacter*) were present in various plant hosts, such as rice [19], *Trifolium repens* L. [56], sugarcane [57], sweetpotato [58], and *Arabidopsis thaliana* [41]. This indicates that these core microbes may be widespread in different plant species. There is no doubt that these common core microbes experience selective pressures and adapt well to life on and within plant tissues. For instance, *Sphingomonas* showed a positive relationship with plant biomass in this study, which may correlation with its ability of producing beneficial secondary metabolites. This study further confirms that there are common principles in the selection of certain microbiotas in different plant niches, in addition to host-driven differences in community assembly [19, 59, 60]. Notably, it was found that, at the ASV level, the generalist microbes that were identified as core inhabitants of rhizosphere microhabitats were more strongly associated with plant functional traits than those found in root microhabitats, and showed a higher ability to predict plant traits (Fig. 5). Indeed, the distinct core microbe recruitment of endospheres was previously shown to be likely the result of an eco-evolution between the microbes and hosts in their arms race for fitness and survival, based on studies of the steady-state root microbiome [61]. These core microbes could be highly adaptive to host environments and may not necessarily be beneficial to the host. In contrast, rhizosphere niches are thought to play a critical role in the control of nutrient availability and plant resource turnover [62]. Thus, our study provides a list of potential candidates for integrating beneficial root microbiomes into modern agricultural practices.

More importantly, although legumes have a group of generalist core microbes overlapping with distinct plant species, the abundance of rhizosphere-indigenous core microbial taxa often differs among host cultivars (genotypes) within a single species [46, 63]. We found that while soybeans with different nodulation phenotypes involved in this study received identical soil environments and were grown under the same greenhouse conditions, unique differential taxonomic profiles were also observed between core microbes (Fig. 5, Fig. S15). The SNS soybean was significantly enriched in *Rhizobiales* in its nodules and had lower bacterial diversity and abundance in rhizosphere core microbes than NNS. These findings suggested that the strategies of the core microbiome assembly of soybean with distinct phenotype use is different [64]. To test this hypothesis, we collected and characterized the metabolic profiles of root exudates at the seedling stage and determined their correlation with core microbiomes (Supplementary Materials B and D). Subsequent analysis revealed significant differences in root exudate metabolic profiles between different cultivars, which were associated with root-associated core microbes (Fig. S16), as substantiated by numerous studies [65, 66]. In addition, although 16 S rRNA gene sequencing is highly valuable as a molecular marker for investigating microbial community diversity, its specificity is limited. The preliminary findings should be validated through comprehensive metagenome profiling and meta-transcriptomic sequencing, accompanied by thorough metabolomics analysis to enhance our comprehension of the associations between both persistent and occasional taxa with plant traits.

## Conclusions

This study provides novel insights into the assembly and potential functioning of root-associated microbes in soybean with different nodulation phenotypes. We found that the core generalist microbiome’s assembly and abundance were correlated with the nodulation phenotype. The supernodulation phenotype soybean enriched *Rhizobiales* in its nodule microhabitats while preferentially enriching N_2_-fixing bacteria in rhizosphere microhabitats, with relatively diminished neutral processes. The dynamics of the generalist core microbiomes of the rhizosphere can partially explain the differences in plant traits. This recruitment is largely in line with the niche-based theory, suggesting the ecological forces of the host and other environmental factors in shaping the bacterial community. These findings contribute to a deeper comprehension and future investigation of synthetic communities and microbiome tools, which are crucial for optimizing plant microbiomes in sustainable agriculture.

### Supplementary information

Supplementary Information

## Data Availability

All 16S rRNA sequencing data for this study were deposited in the NCBI Sequence Read Archive (SRA) database under accession number SRP400755. Re-sequencing data of this study have been deposited in the NCBI SRA database under accession numbers SRR22556434 (SNS) and SRR22556435 (NNS). The sequencing data of Chinese Zhonghuang 13 and American William 82 were obtained from the GSA repository (CRR031689) and NCBI SRA database as a reference (SRR4253028), respectively.
