# Supplementary material for: Nodulation number tempers the relative importance of stochastic processes in the assembly of soybean root-associated communities
Source: ISME Commun. 2023 Aug 28;3:89. doi: 10.1038/s43705-023-00296-8 (PMC10462722; doi:10.1038/s43705-023-00296-8)
Supplement: Supplementary file 1 — Supplementary Information [file 43705_2023_296_MOESM1_ESM.pdf]

Supplementary information

Nodulation number temper the relative importance of stochastic processes in the assembly of soybean root-associated communities

A. Information of two cultivars

The ecological forces that structure microbial communities were surveyed using two soybean (*Glycine max*) cultivated varieties with obvious differences in nodulation abilities. Heihe 43, bred by the Heihe Branch of Heilongjiang Academy of Agricultural Sciences, has approximately 75 cm plant height, lanceolate leaves, gray pubescence, purple flowers, a sub-indeterminate podding habit, and only one main stem with no distinct branches. The seed of Heihe 43 is shiny, round, yellow, and has a light-yellow hilum (Fig. S1A). The hundred seed weight (HSW) is about 18 g, the protein content is about 42%, and the fat content is about 19%. It takes about 115 days from seedling emergence until final harvest at maturity, with 2150 °C accumulated average temperature (over 10 °C). According to the ripening, Heihe 43 is most suitable for planting in the Fourth Accumulation Temperature Belt of Heilongjiang Province. HZ 9009, bred by Prof. Shaodong Wang of Northeast Agricultural University, has approximately 70 cm plant height, lanceolate leaves, gray pubescence, purple flowers, sub-indeterminate podding habit, and a single stem with few branches. The seed of HZ 9009 is shiny, round, yellow, and has a light-yellow hilum (Fig. S1B). The hundred seed weight (HSW) is about 28 g, the protein content is about 45%, and the fat content is about 19%. It takes about 110 days from seedling emergence until final harvest at maturity, with 2100 °C accumulated average temperature (over 10 °C). According to the ripening, Heihe 43 is most suitable for planting in the Fourth Accumulation Temperature Belt of Heilongjiang Province.

To identify genetic distance between varieties, we applied Simka (v.1.5.3) *k*-mer genome comparison analysis (ab-jaccard distance) on re-sequencing data. In total, we obtained approximately 42.3, 40.6, 39.3 and 9.5 Gb raw reads for HZ 9009, Heihe 43, Zhonghuang 13 and Williams 82, respectively. After quality control filtering, 36.5, 34.5, 31.3 and 5.7 Gb clean reads were obtained.

Compared with Zhuanghuang 13 and William 82 with large genetic variation, the distance between HZ 9009 and Heihe 43 is very close, indicating that the genetic variation between them is relatively small (Fig. S1b).

B. Potential molecular mechanisms underlying the variation in nodule number

The control of nodule number involves intricate molecular mechanisms and the participation of key regulatory genes. Among these, the autoregulation of nodulation (AON) pathway plays a vital role in balancing nodule development to maintain optimal nitrogen fixation efficiency. The currently known AON pathway includes four parts: soil NO_3^- signal and Rhizobium signal recognition and transmission, CLE-SUNN is the negative regulation pathway, CEP-CRA2 is the positive regulation pathway and the miR2111/TML module regulates nodule formation and development [1,2]. Based on the gene information reported in the literature associated with nodule number, we performed alleles mapping on the draft genome of HZ9009 to identify potential variant sites. Fortunately, we have successfully identified a candidate gene, *GmNARK*. We employed blastn and collinearity analysis to locate and predict the *GmNARK* alleles in HZ9009, using the *GmNARK* alleles from Wm82 and HH43 as references. The genome version of Wm82 used was Wm82.a2.v1, downloaded from the Phytozome website (https://phytozome-next.jgi.doe.gov/), with the *GmNARK* allele ID being *Glyma.12G040000* and the ID of the main transcript's CDS sequence being *Glyma.12G040000.1*. For HH43, the genome version was SoyC13.v1, obtained from the SoyOmics website (https://ngdc.cncb.ac.cn/soyomics), with the *GmNARK* allele ID being *SoyC13_12G036700* and the ID of the main transcript's CDS sequence being *SoyC13_12G036700.m2*. The results revealed that the *GmNARK* gene in HZ9009 was also located on chromosome 12 (Chr12:2851630-2855465). At the CDS level, the sequences of Wm82 and HH43 were identical, while two variant sites were identified in the CDS sequence of HZ9009. One

SNP was a synonymous mutation (G-A) at position 1155 bp, while the other SNP was a non-synonymous mutation (A-T) at position 1816 bp, resulting in a premature stop codon and premature termination of translation. Figure R1 illustrates the alignment of the two variant sites in the *GmNARK* gene among three cultivars.

The *GmNARK* gene expressed in the leaves is a key negative regulatory factor in the AON pathway responsible for long-distance communication [3]. Mutations in this gene have been found to significantly increase the number of soybean nodules [4]. Previous studies have reported soybean cultivars, such as En6500 and Sakukei 4, harboring the same nonsense mutation site as found in HZ9009. These cultivars exhibit a premature termination of translation in the *GmNARK* protein, resulting from a nucleotide mutation at the 606th amino acid residue, where the base sequence changes from AAA to TAA [5]. Furthermore, studies on these *GmNARK* gene mutant varieties have demonstrated that, compared to the controls, these mutants exhibit significantly higher nodule numbers, heavier hundred seed weight, and relatively smaller plant stature [6]. These findings closely align with the phenotypic differences observed between HZ9009 and HH43. Therefore, we hypothesize that this molecular alteration serves as the molecular basis for the super-nodulating phenotype observed in HZ9009. However, we acknowledge that further investigations, including the recovery of mutations and additional molecular experiments, are necessary to definitively determine whether the premature termination mutation is the underlying cause of the super-nodulating phenotype observed in HZ9009.

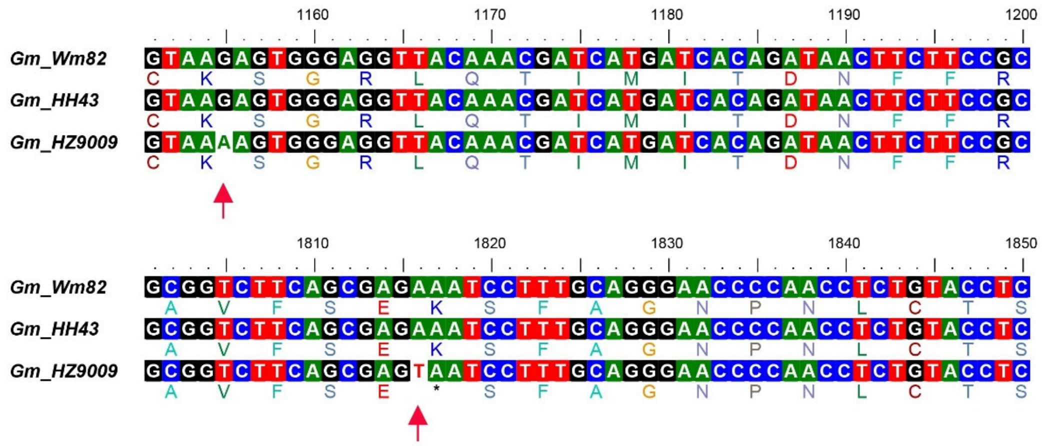

Figure R1. Multiple Sequence Alignment of *GmNARK* Gene CDS Regions in Different Soybean Varieties

C. Materials and methods

Soil properties and experimental design. The physical and chemical properties of the topsoil were as follows: pH, 6.38; organic matter, 25.25 g kg^{-1}; available P, 20.55 mg kg^{-1}; available K, 221.70 mg kg^{-1}; and soil texture, silty loam. The test soil was sampled, transported back to the greenhouse and then homogenized and sieved with a 2-mm sieve. Seeds of these two cultivated soybeans varieties were surface-sterilized with 75% ethanol for 3 min, thoroughly rinsed in deionized water, and germinated in the dark. After that, the soybean seeds were planted in pots (19 cm × 18 cm) containing the abovementioned soil (3.0 kg). Each pot was sown with six soybean seeds in a triangular pattern and placed in a greenhouse under long-day conditions (16-h photoperiod, 20 ℃/28℃, night/day). At the two-leaf stage, thin the plants to 3–4 pot^{-1} and water with tap water as needed. Sample collections were performed at the soybean seedling (V2), flowering (R2), podding (R4), and mature (R8) stages in 2021 (Fig. S2). At the same time, several physiological and morphological features were determined to quantify the functional properties of the plants. Plant dry biomass, plant height, plant C:N ratio, and root nodule number were derived from direct measurements. Tissue nitrogen and carbon contents were

measured with an elemental analyzer (EA3000; Euro Vector, Italy).

For each time point, the rhizosphere soil, root, and nodule samples were collected according to our previous methods, with modifications. Briefly, the plant roots were carefully shaken to remove the soil particles that were not tightly adhered. The remaining soil (approximately 1 mm thick still attached to root) was carefully collected by vortexing the roots in phosphate-buffered saline (PBS) and centrifuging the soil suspension to form a rhizosphere soil sample for each pot. For sampling the root endosphere sample, the cleaned root was placed in fresh buffer for three sonication procedures (30 s at 42 Hz, 30 s pause) to remove tightly adhering soil particles. Root nodules were collected after root sonication. The plant samples were wrapped in tinfoil and then frozen in liquid nitrogen. Unplanted soil samples were also collected for the controls (bulk soil). There were 3–5 replicates of 3–4 seedlings each measured for each treatment. Within each root and rhizosphere replicate, individual samples were extracted without pooling. Each bulk soil replicate represented a pool of three soil samples. These fresh samples were stored at -80 °C prior to DNA extraction.

In addition, Canonical analysis of principal coordinates (CAP) on the Jaccard metrics was also performed to measure the variance attributable to nodule number. The variance partitioning and significance analysis for experimental factors were conducted by applying Vegan's permutest() function to the CAP model. In the soil data analysis, bulk soil samples were excluded from the CAP analysis due to their potential confounding effect within the Cultivar factor.

Collection and characterization of root exudates. Soybean root exudates were collected from two soybean cultivars at the vegetative growth stage (V2) due to the pivotal role of root exudates during this stage in shaping rhizosphere microbiomes [7-9]. Briefly, 35-day old seedlings (see above for seedling growth) were gently washed with sterile water to remove the surface-adhering soils before

being transferred to conical flasks containing 260 mL of sterilized MilliQ water for a 3-hour period of continuous secretion under natural ambient light. Wrap the conical flask in Aluminum foil to protect from light. Three biological replicates were collected for each cultivar, and within each replicate, three seedlings were pooled to obtain exudates from a total of 9 seedlings per cultivar. The root exudates were collected and filtered through 0.45 μm pore size nylon filters (Millipore, MA) to eliminate any border-like cells and root sheathing. After being filtered, the exudates were subjected to freeze-drying (Labconco, MO) and subsequently stored at −80°C for further analysis. The samples were thawed and mixed for 30 seconds using a vortexer, then transferred to numbered 50 mL centrifuge tubes and frozen overnight at -80 ℃ before undergoing vacuum freeze drying. After freeze-drying, add 30 times the concentration of a 70% methanolic internal standard extract. Vortex for 15 minutes, sonicate in an ice water bath (KQ5200E) for 10 minutes at 12,000 r/min, and centrifuge at 4°C (5424R, Eppendorf) for 3 min. Decant the supernatant through a microporous membrane with a pore size of 0.22 μm and store it in an injection vial for LC-MS/MS detection.

Detection, identification and quantification of the sample extracts were analyzed using the UPLC-ESI-MS/MS system (UPLC, ExionLC™ AD) and Tandem mass spectrometry system. The UPLC analytical conditions were as follows: column, Agilent SB-C18 (1.8 μm, 2.1 mm × 100 mm). The mobile phase consisted of solvent A (pure water with 0.1% formic acid) and solvent B (acetonitrile with 0.1% formic acid). The measurements were performed using a gradient program that started with 95% A and 5% B. Within 9 minutes, the composition was linearly changed to 5% A and 95% B, which was maintained for one minute. Subsequently, a composition of 95% A and 5.0% B was adjusted within 1.1 min and maintained for 2.9 min, with a flow rate of 0.35 mL/min, column oven temperature set at 40°C, and an injection volume of 2 μL. The effluent was then connected alternatively to an ESI-triple

quadrupole-linear ion trap (QTRAP)-MS. The ESI source was operated at 500°C with an IS voltage of 5500 V (positive ion mode)/-4500 V (negative ion mode). Gas I, Gas II, and curtain gas were set at 50, 60, and 25 psi respectively. Collision-activated dissociation was set to high for QQQ scans conducted as MRM experiments using medium collision gas (nitrogen). Declustering potential (DP) and Collision energy (CE) optimization was performed for each individual MRM transition, followed by further refinement of DP and CE. The monitored MRM transitions were specific to the metabolites eluted in each period. The peak area of each identified compound in the root exudate fraction underwent a log transformation for data analysis. The Pearson correlation between ASV abundances and the exudate fraction was computed using R Studio. The abundance of each ASV in each sample was log2 transformed and standardized to have a mean of 0 and a standard deviation of 1.

D. Basic survey of 16S datasets

After removal of plant-derived reads and filtering, a total of 5,912,481 nonchimeric sequences were yielded with a median read count per sample of 60,832 (range 25,090–134,209; Table S2). For each sample, 23,730 random sequences were subsampled to a normalized read depth across all samples based on the 95th percentile of the lowest sampling depth. All further results are based on this rarefied read depth. The rarefaction curves of different samples based on the ASV numbers are shown in Supplementary Fig. S3-5.

D. Exudate metabolic profiles

A total of 917 compounds were detected in the root exudates recovered from the two soybean cultivars, of which were categorized into seven chemical classes, including alkaloids, flavonoids, lignans and coumarins, phenolic acids, quinones, tannins and terpenoids. PCA ordination showed that the root exudate profiles from supper-nodulating and normal nodulating soybean were significantly separated from each other (Fig. S17a), with the first two principal components explaining 61.91% (44.45% and 17.46%) of the total variation. Accordingly, the cluster dendrogram also distinguished two groups (Fig. S17b) which is consistent with the PCA. The types of chemicals were the same across the two soybean cultivars, but distinct differences between the abundance of some compounds were detected. In total, 30 enriched and 192 depleted compounds were identified, and mainly categorized into flavonoids (39.44%) and phenolic acids (37.50%). Specially, super-nodulating soybean selected a higher quantity of 7-methoxycoumarin, kaempferol-3-o-neohesperidoside and epipinoresinol than soybean with normal nodulation phenotype (Fig. S17e), with the lower quantity of paeonicluside and kaempferol-3-o-(6"-rhamnosyl-2"-glucosyl) glucoside (camelliaside A).

Procrustes analysis showed a significant association between root-associated core microbes and root exudate profiles (Rhizosphere: M^2 = 0.037, P = 0.018, Root: M^2 = 0.391, P = 0.042). Thus, to identify the major metabolite factors in our data set, we correlated dissimilarities of core microbes' composition with those of metabolites. Overall, alkaloids and phenolic acids were the strongest correlates of both rhizosphere and root core microbes' structure, while no significant correlation was found for terpenoids. Flavonoids were only significantly correlated with the structure of core microbes inhabiting rhizosphere.

E. Supplementary figures

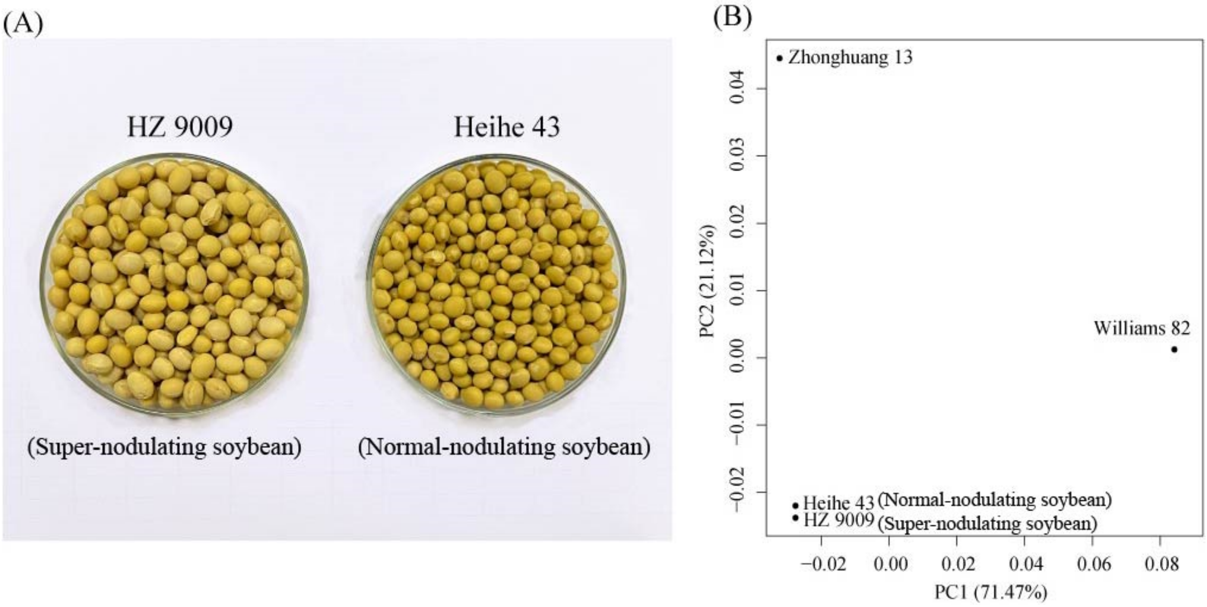

Fig. S1 Simka *K*-mer genome comparison analysis of plant materials. (A) Seeds of super- and normal-nodulating soybean; (B) PCA plot of soybean genetic variation.

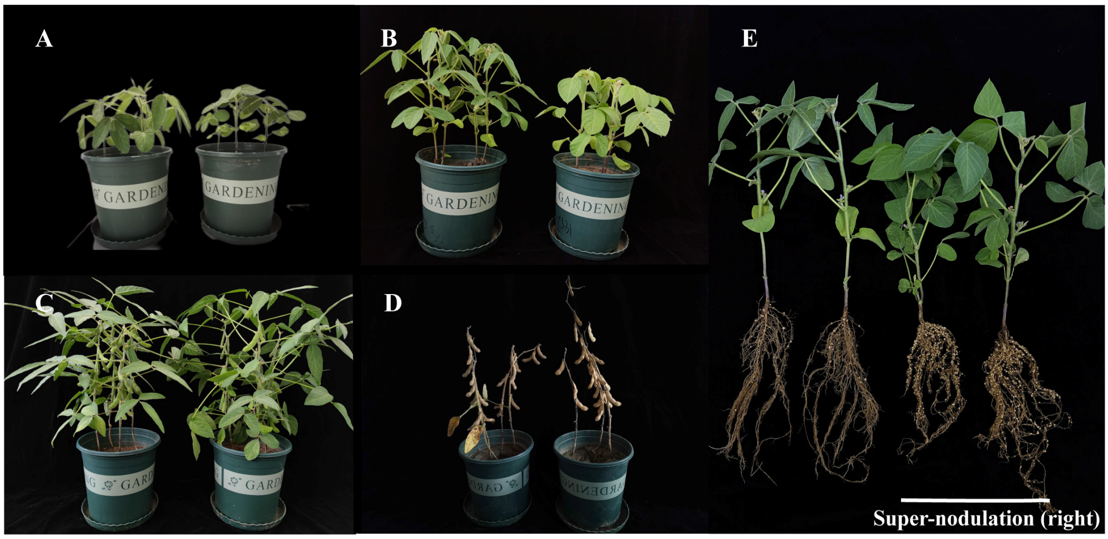

Fig. S2 The representative growth status of super-nodulating soybean (SNS) and normal nodulating soybean (NNS) across four plant developmental stages. Growth status of the two soybean cultivars in seedling stage (A), flowering stage (B), full pod stage (C), and mature stage (D). Nodulation ability of the two soybean cultivars in flowering stage (E).

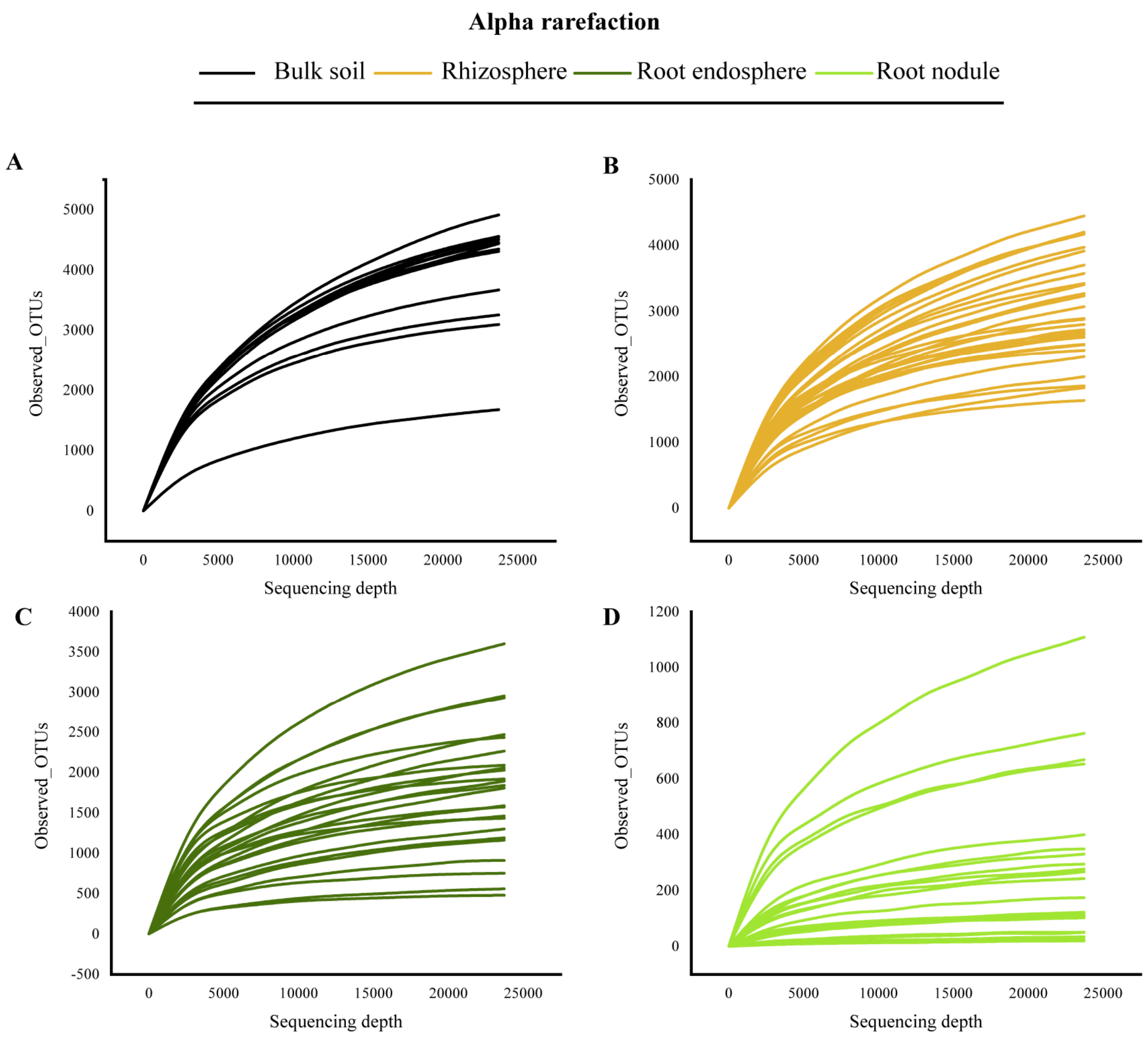

Fig. S3 Rarefaction curves for Observed OTUs of individual soybeans per root compartment (A, bulk soil; B, rhizosphere; C, root; and D, root nodule).

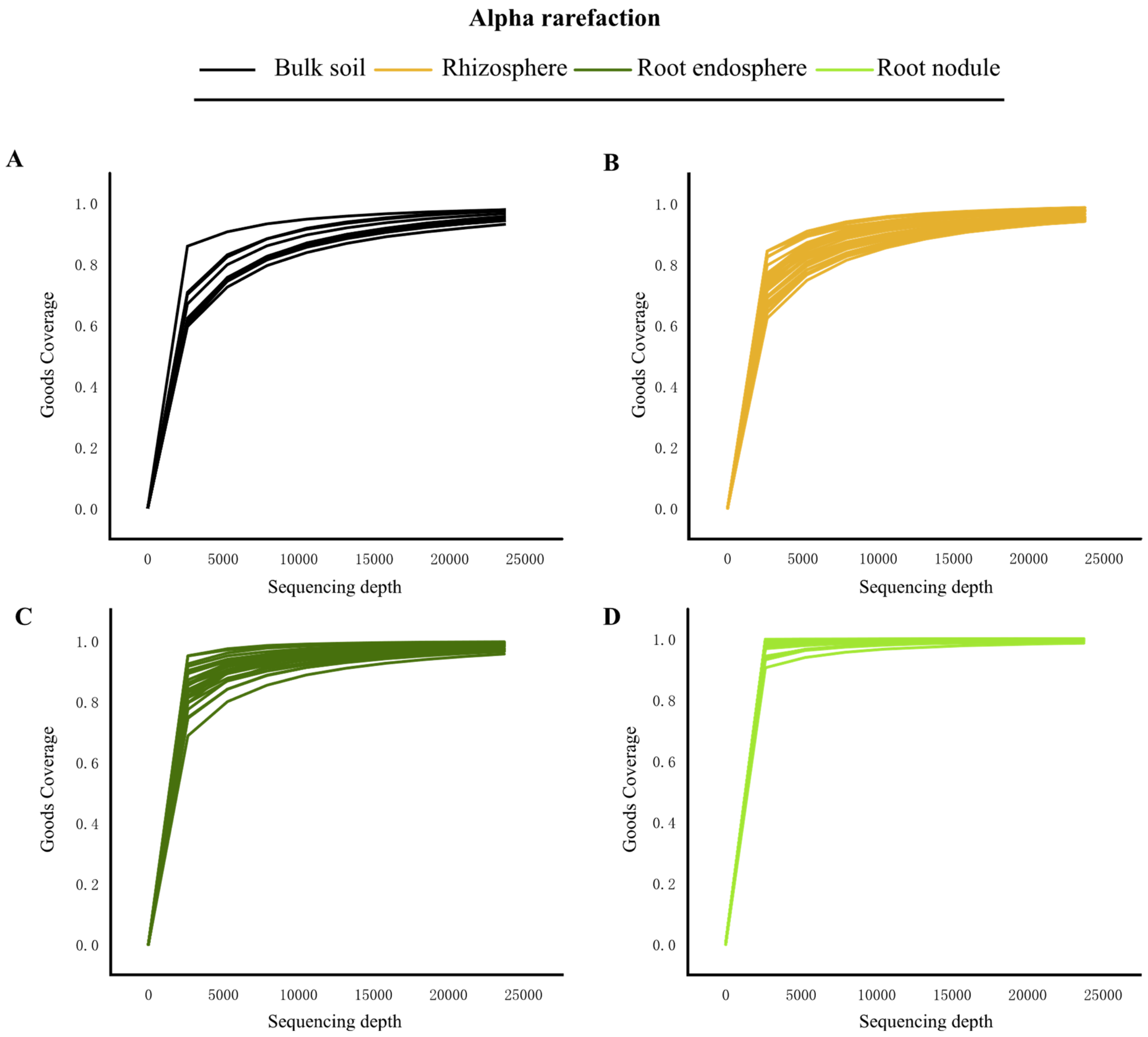

Fig. S4 Rarefaction curves for Goods coverage of individual soybeans per root compartment (A, bulk soil; B, rhizosphere; C, root; and D, root nodule).

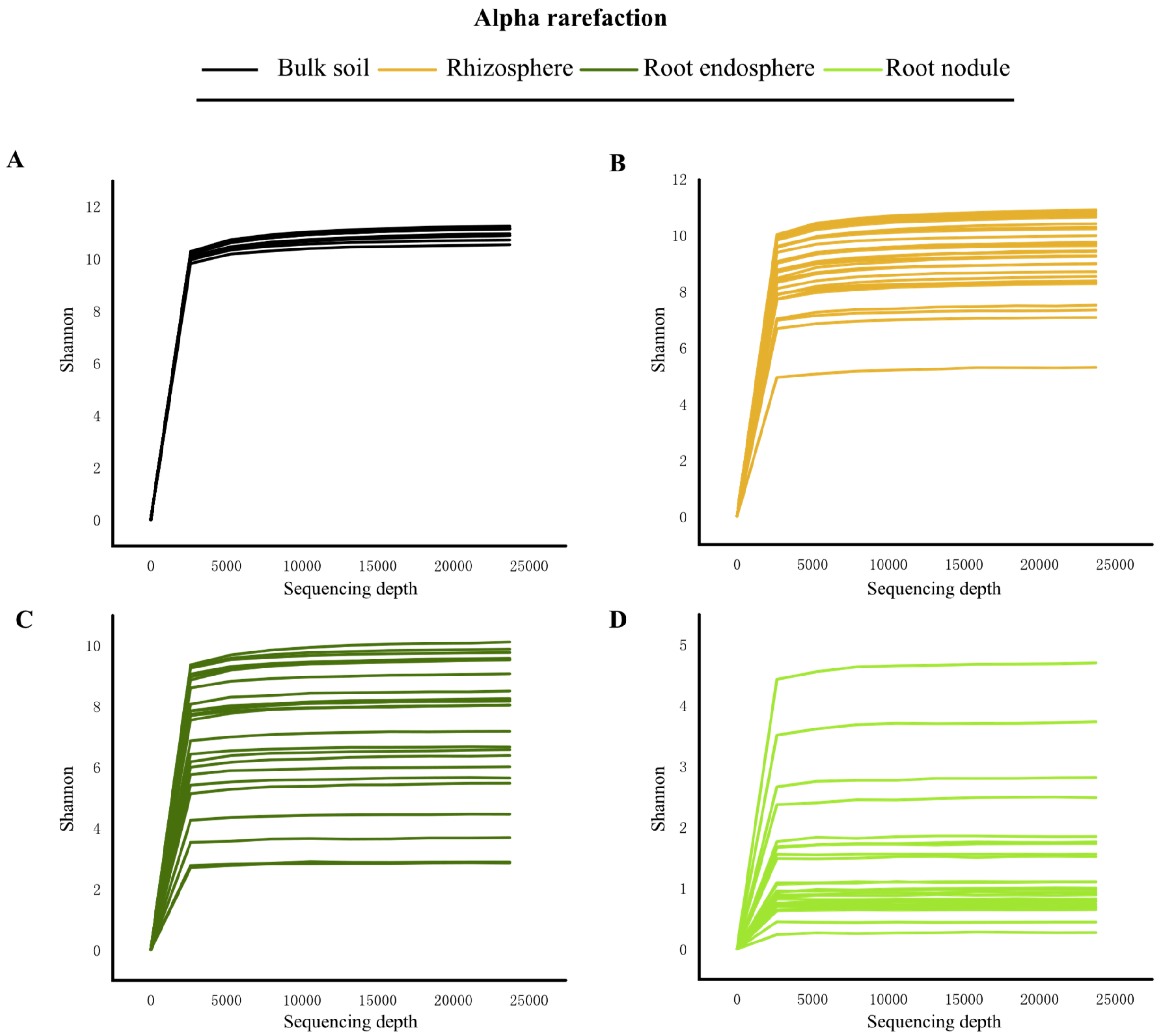

Fig. S5 Rarefaction curves for Shannon of individual soybeans per root compartment (A, bulk soil; B, rhizosphere; C, root; and D, root nodule).

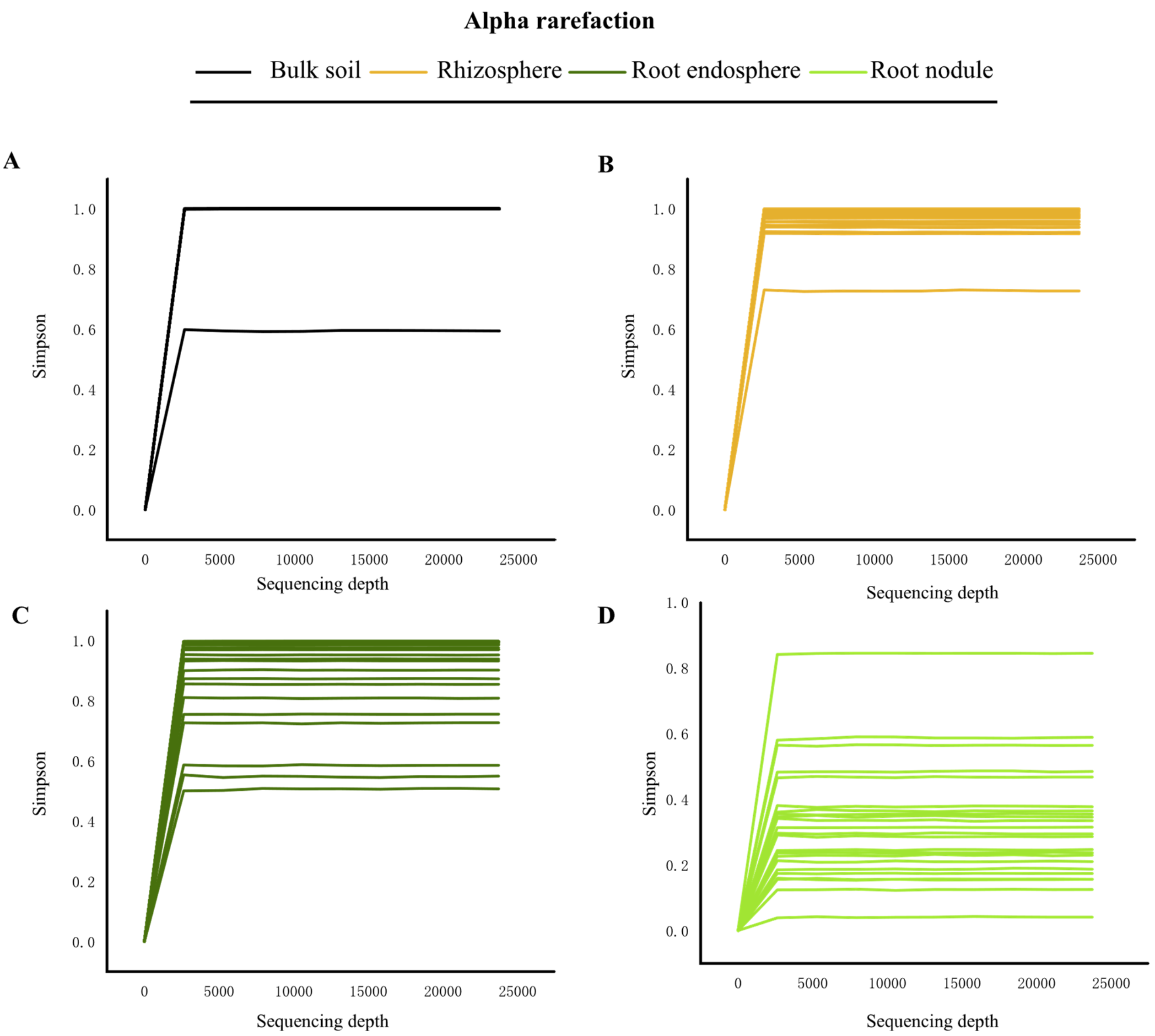

Fig. S6 Rarefaction curves for Simpson of individual soybeans per root compartment (A, bulk soil; B, rhizosphere; C, root; and D, root nodule).

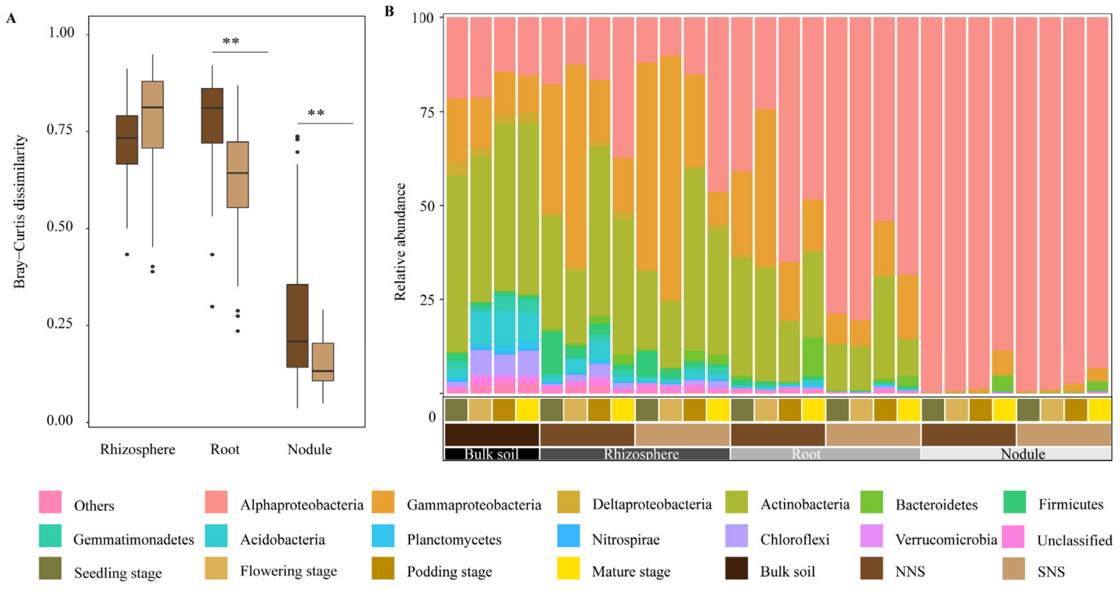

Fig. S7 Temporal dynamics of diversity and distribution patterns of root-associated microbiomes. (A) Differences in beta-diversity among for each microbial group. $**P < 0.01$. (B) Taxonomic composition of bacterial microbiomes. The ASVs of Proteobacteria have been replaced by those at the subclass level (alpha, delta and gamma). *SNS*, super-nodulating soybean; *NNS*, normal nodulating soybean.

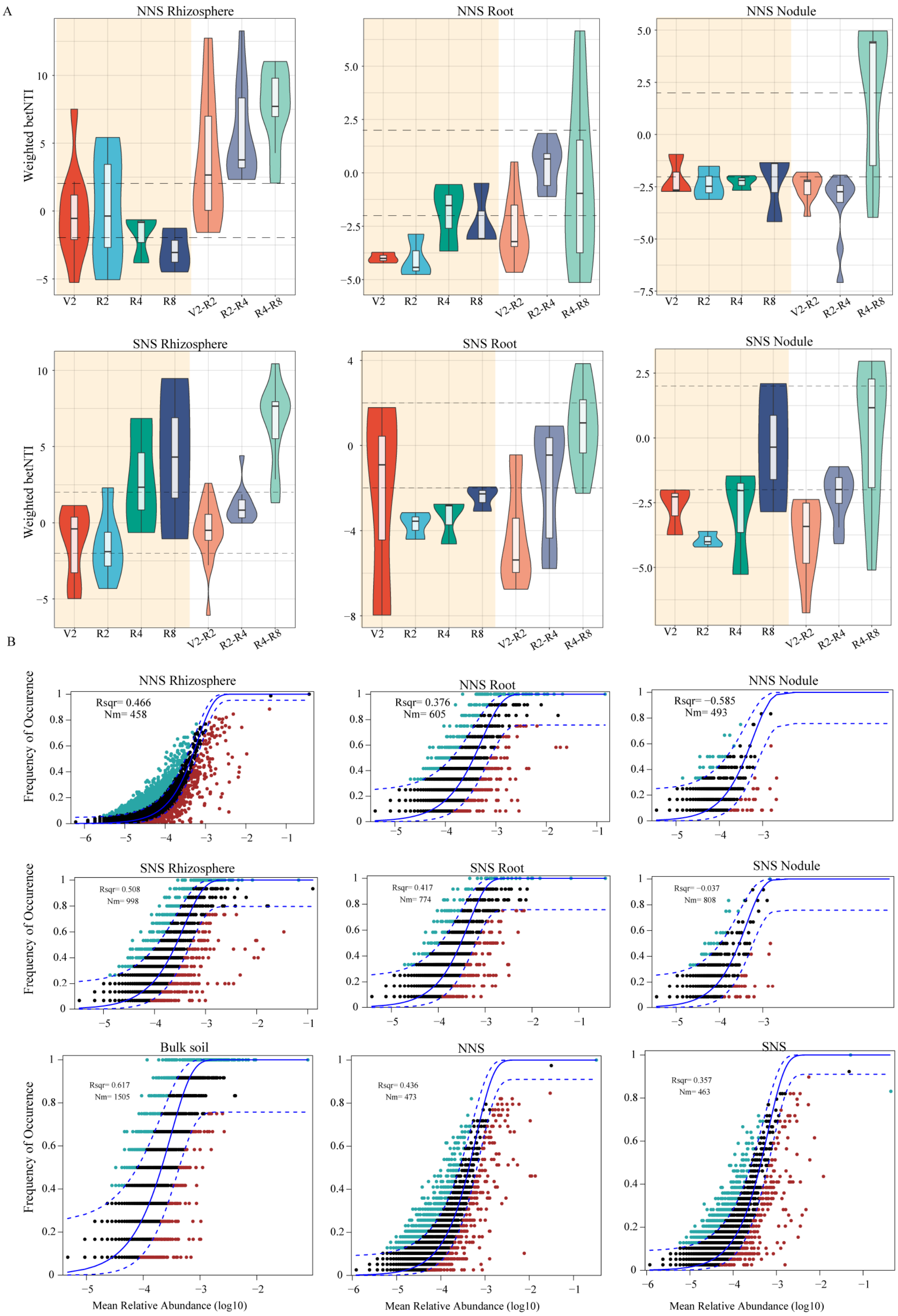

Fig. S8 Deterministic and stochastic processes in microbiome assembly. (A) β-Nearest taxon index (βNTI) values of bacterial communities. The βNTI value was estimated using null model, and |βNTI| greater than 2 represents dominant determinism in regulating community assembly. Otherwise, it

represents dominant stochasticity in regulating community assembly. (B) The neutral model (NCM) fitting of bacterial communities inhabiting in different root-associated microhabitats. The solid blue lines indicated the best fit to the NCM and the dashed blue lines represent 95% confidence intervals around the model prediction. ASVs that occur more or less frequently than predicted by the NCM are shown in different colors. Nm indicates the metacommunity size times immigration, R2 indicates the fit to this model. *SNS*, super-nodulating soybean; *NNS*, normal nodulating soybean; *V2*, second trifoliolate stage; *R2*, full bloom stage; *R4*, full pod stage; *R8*, full maturity.

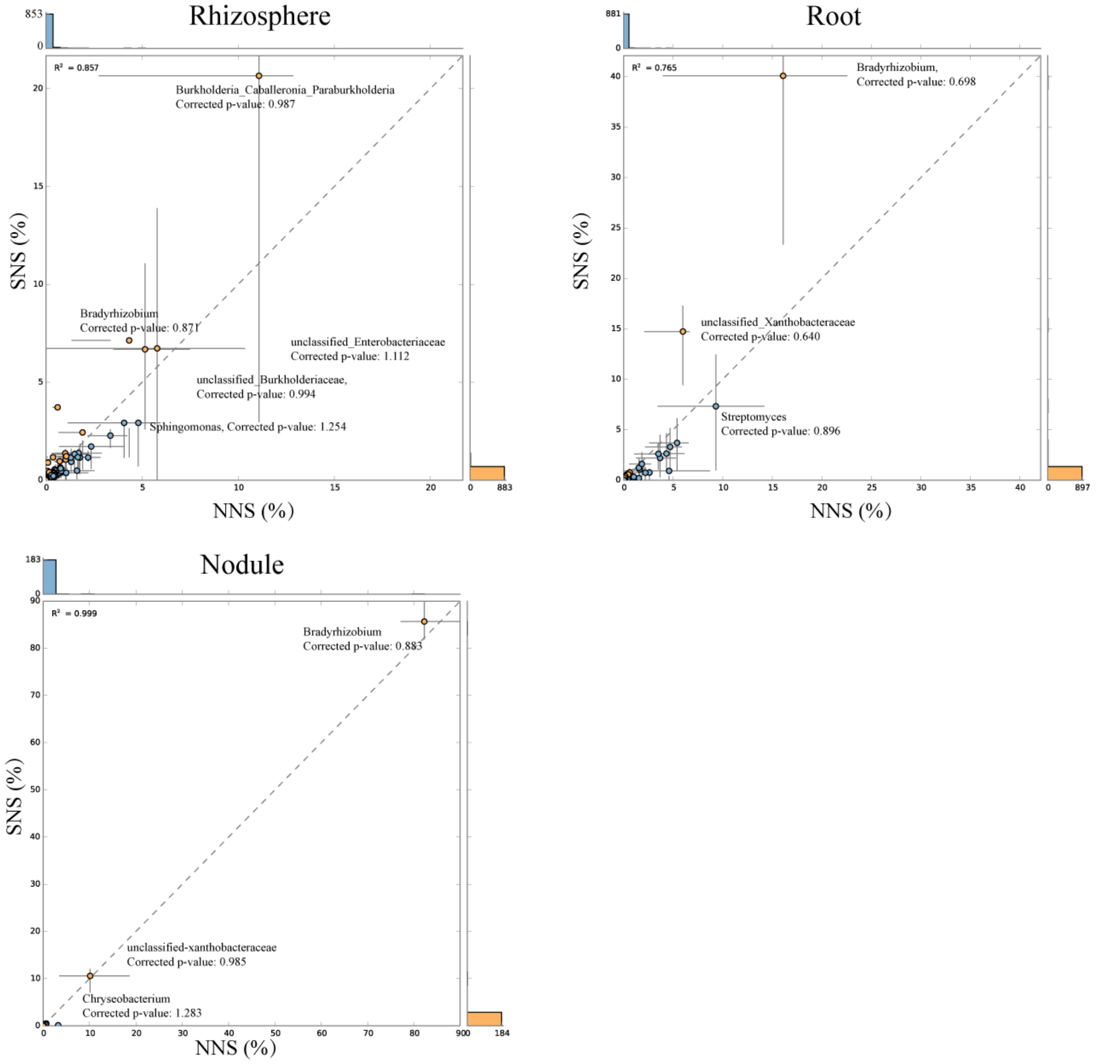

Fig. S9 Microbes influenced by nodule number in soybean rhizospheres, root and nodule. Microbes differentiating NNS and SNS were compared using STAMP software based on genus abundance. Corrected P values were calculated using the two-sided Welch's t test with the Benjamini-Hochberg FDR correction. *STAMP*, statistical analysis of taxonomic and functional profiles; *FDR*, false discovery rate; *SNS*, super-nodulating soybean; *NNS*, normal nodulating soybean.

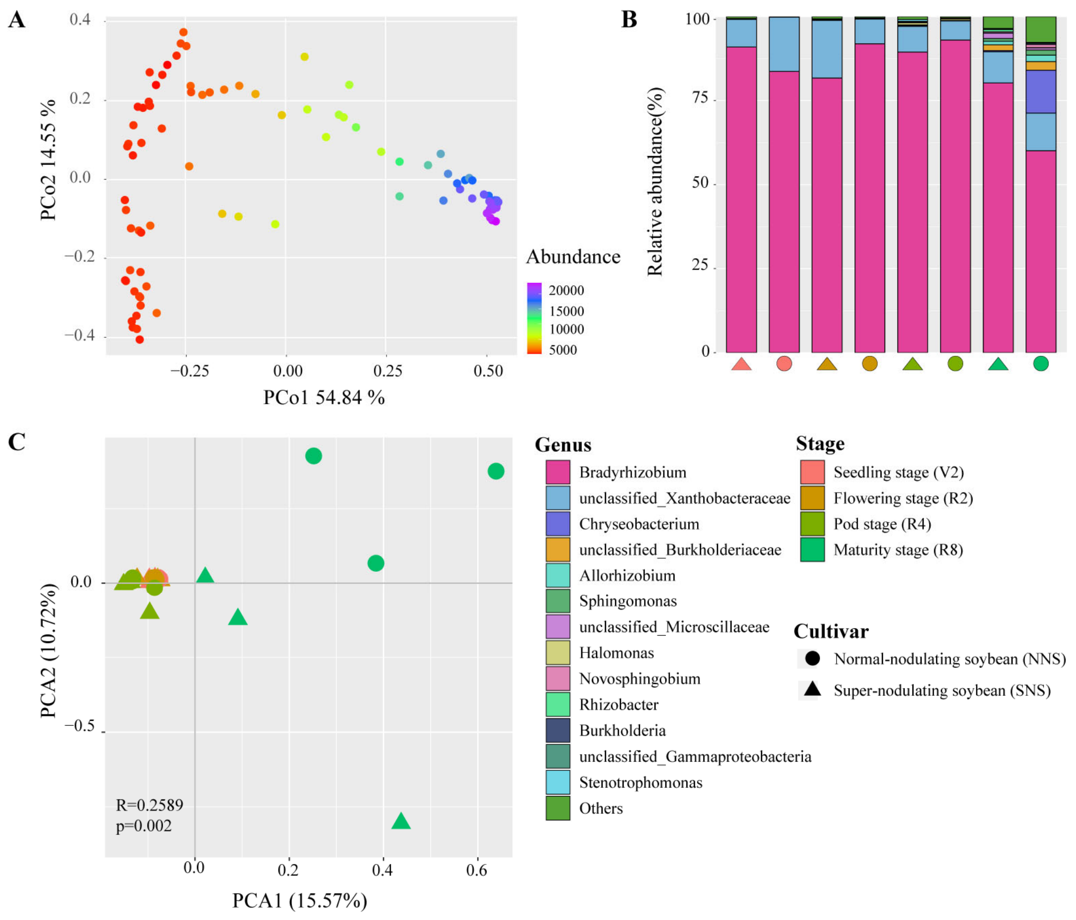

Fig. S10 Shifts in the microbiota during plant development stages are associated with *Bradyrhizobium*. (A) Principal coordinates analysis (PCoA) of the Bray-Curtis dissimilarity between overall root-associated samples colored by root *Bradyrhizobium* abundance. (B) Bar plots of the relative abundance of the top 13 genera throughout the plant growth stages in nodule microhabitats. (C) Principal Component Analysis (PCA) of the composition of ASV members belonging to *Bradyrhizobium* in both super-nodulating soybean and normal nodulating soybean.

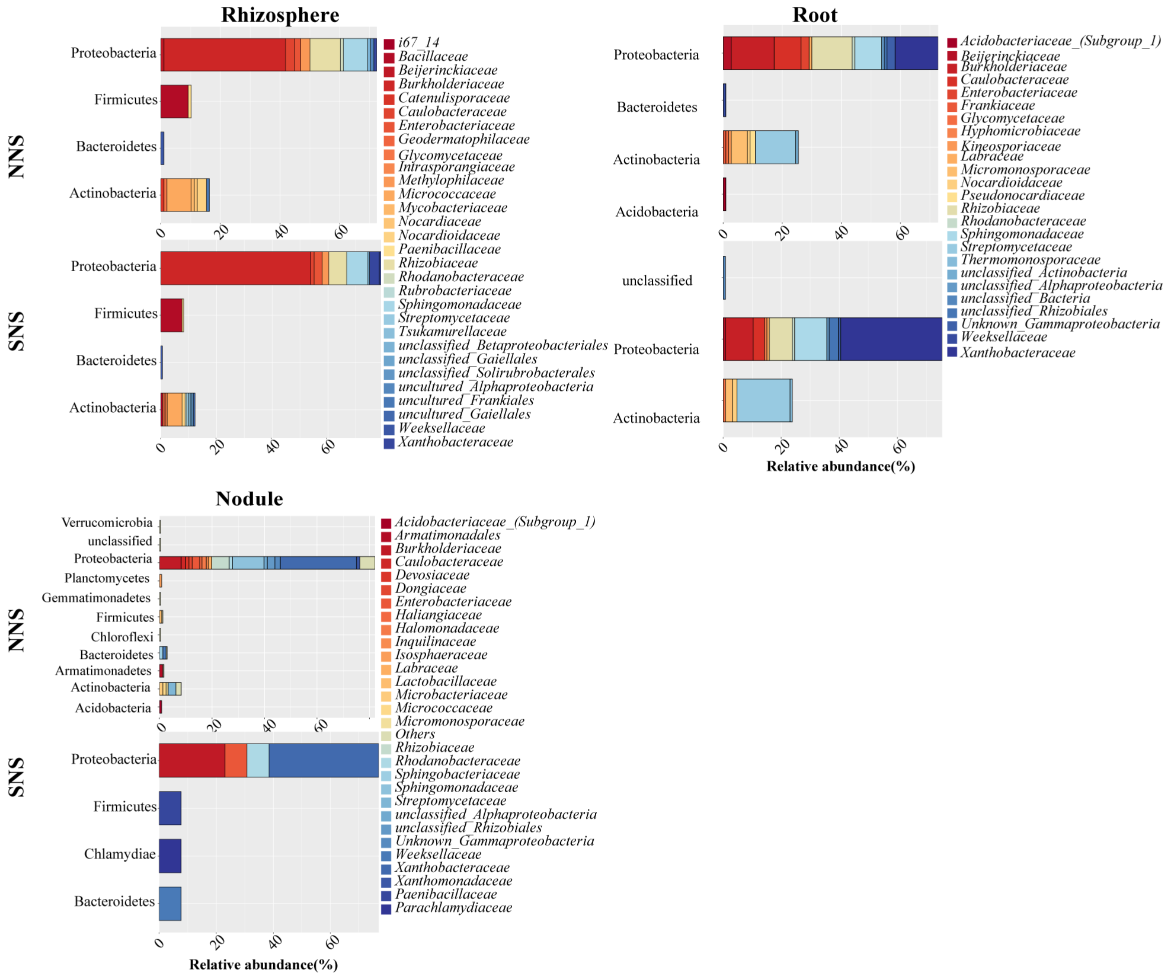

Fig. S11 Number of ASVs and the phyla and families enriched across rhizosphere, root and nodule microhabitat. *SNS*, super-nodulating soybean; *NNS*, normal nodulating soybean.

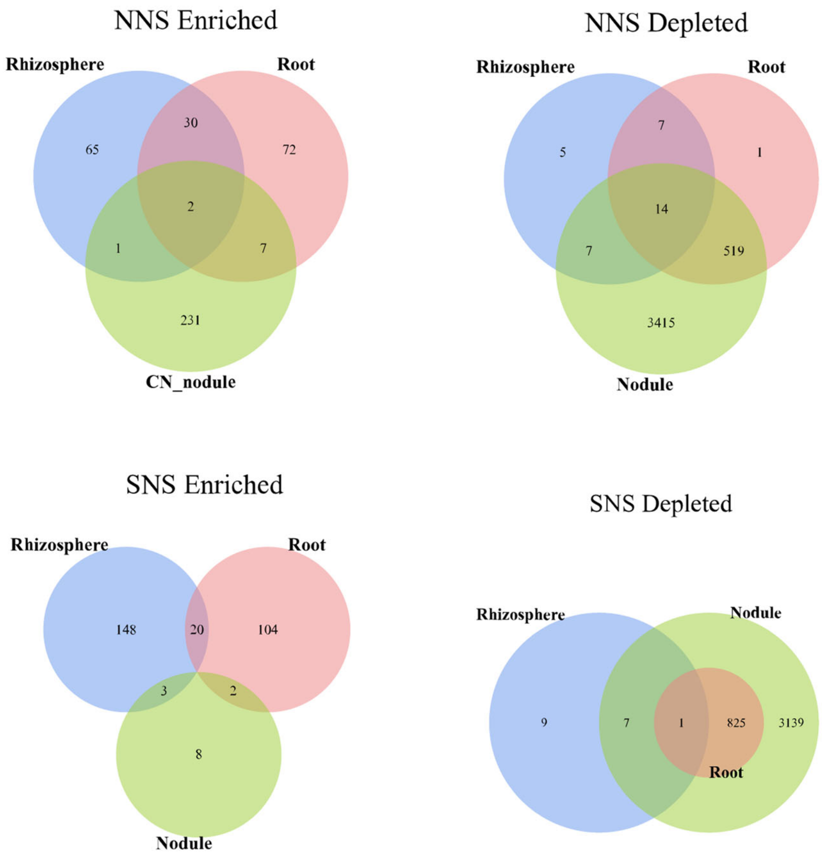

Fig. S12 Rhizo-microhabitats are enriched and depleted for certain ASVs.

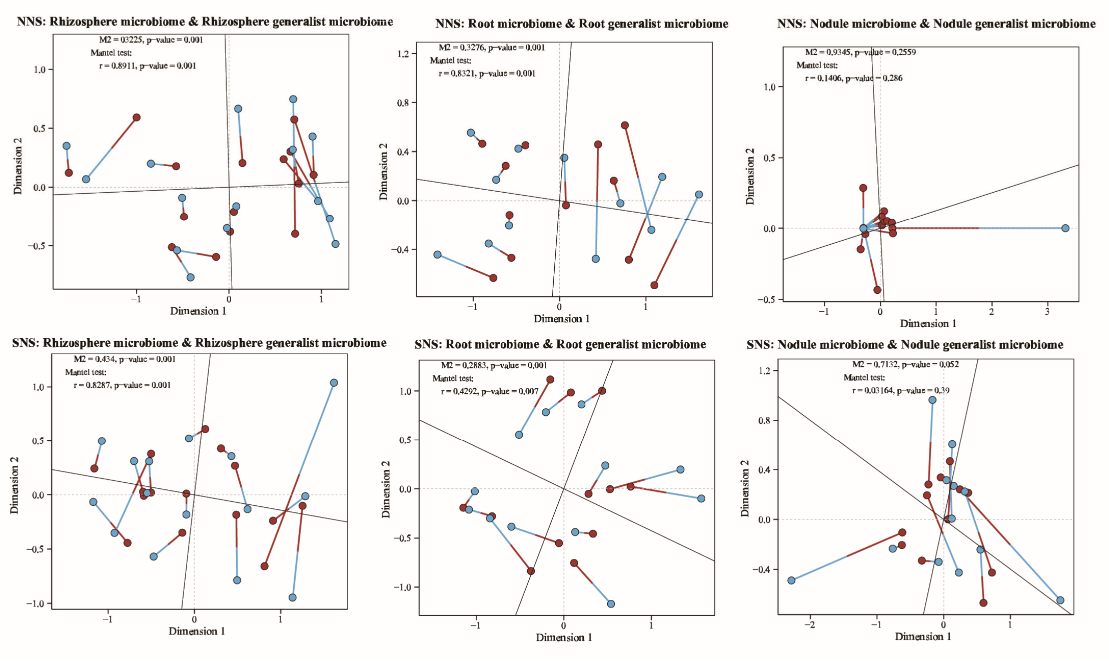

Fig. S13 Procrustes correlation between the total microbiota and generalist microbiota. *P* values were determined from 999 labelled permutations. *r* indicates the correlation in a symmetric Procrustes rotation. M^2 represents the sum of squared distances between matched sample pairs.

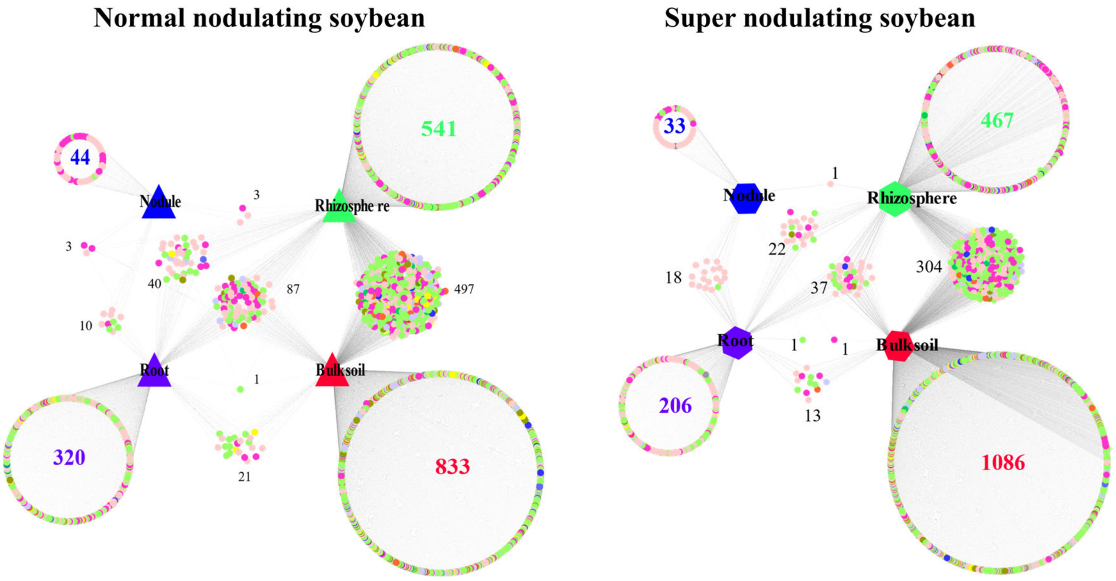

Fig. S14 Bipartite association networks of generalist microbes implicated across root-associated microhabitats.

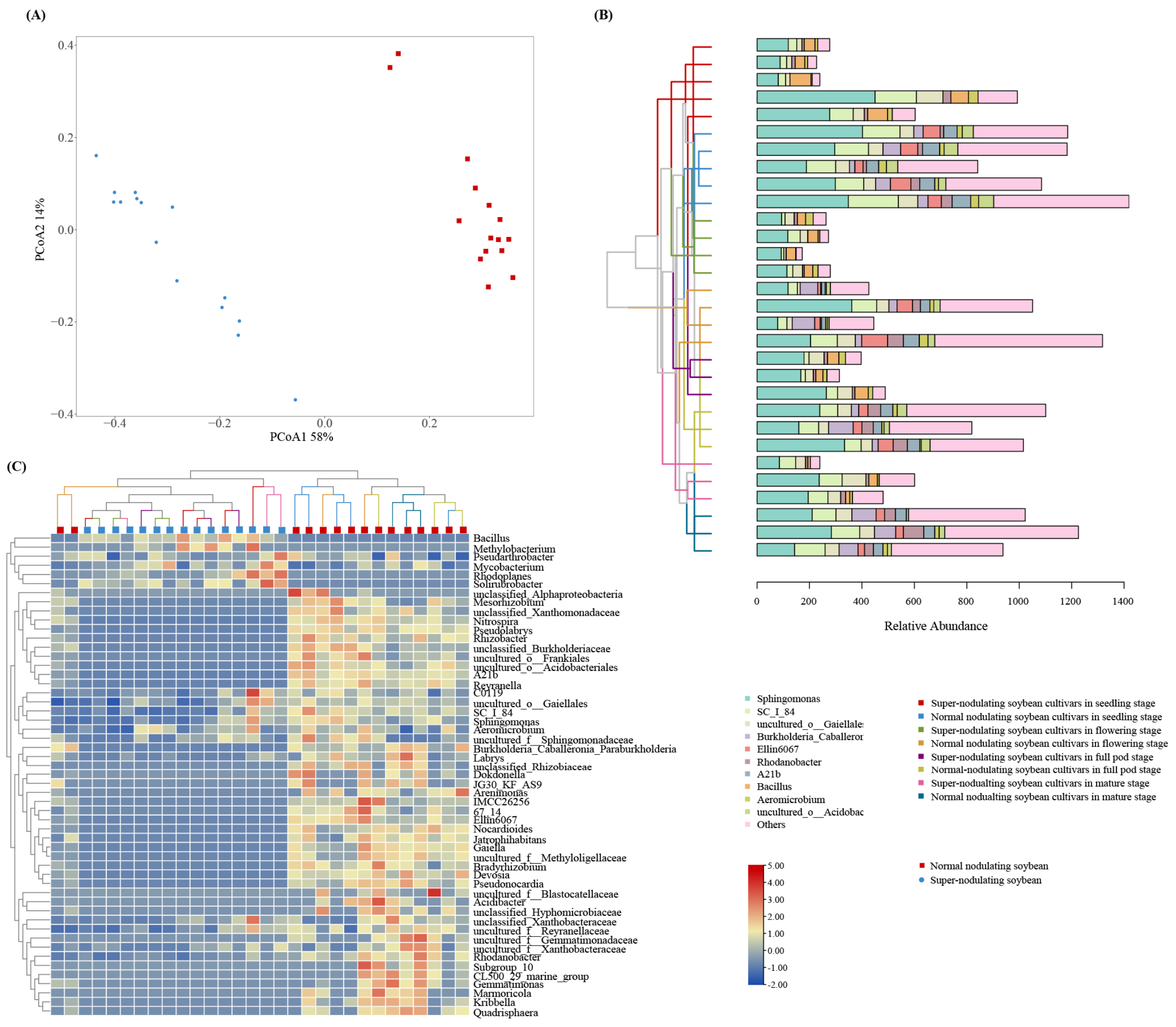

Fig. S15 Core generalist bacterial communities' selection in the rhizosphere microhabitats of soybean with different nodulation phenotypes. (A) Principal coordinate analysis (PCoA) plots for visualization of Bray-Curtis distances among the bacterial communities. (B) The relative abundance levels of dominant genus. (C) A heatmap showing core generalist bacterial communities that were differentially abundant across soybean cultivars.

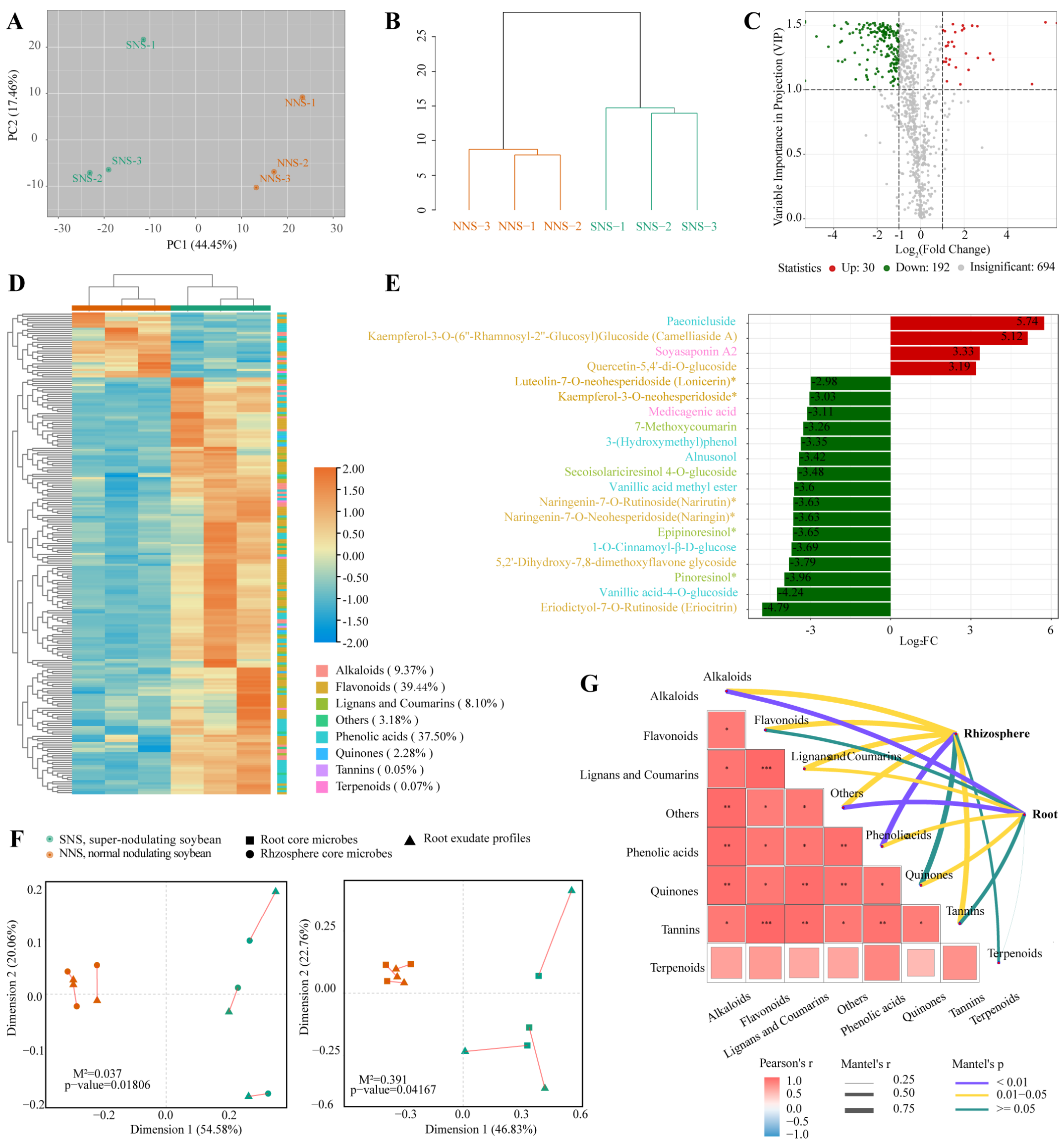

Fig. S16 Differential abundance of root exudate composition and associated with core microbes.

(A) PCA analysis of root exudates of both super-noudating soybean and normal nodulating soybean. (B) Cluster dendrogram of root exudates of both super-noudating soybean and normal nodulating soybean. (C) Volcano plot of metabolites identified in root exudates. The volcano plot illustrates the fold change of each metabolite, and the differential metabolites were determined based on VIP (VIP > 1) and absolute Log2FC (|Log2FC| ⩾ 1.0). (D) Heat map of the differential metabolites identified in root exudate samples. (E) Bar diagram of the top 20 significantly enriched up- and down-regulated metabolites. The color of each text reflects the class of metabolites. (F) Procrustes correlation between

the core generalist microbiota and the root exudate. P values were determined from 999 labelled permutations. M2 represents the sum of squared distances between matched sample pairs. (G) Pairwise comparisons of metabolites are shown, with a color gradient denoting Pearson's correlation coefficient. The compositions of root-associated core microbes inhabiting in rhizosphere and root microhabitat were related to each metabolite by partial Mantel tests. Edge width corresponds to the Mantel's statistic for the corresponding distance correlations, and edge color denotes the statistical significance based on 9,999 permutations. *SNS*, super-nodulating soybean; *NNS*, normal nodulating soybean. Medicagenic acid is short for Medicagenic acid-3-O-glucuronide-28-O-xylosyl(1,4)-[apiosyl (1,3)]-rhamnosyl (1,2)-arabinoside.

F. Supplementary tables

Table S1 Plant functional traits of different soybean cultivated varieties

**Cultivar**	**Stage**	Aboveground N content	Aboveground C content	Underground N content	Underground C content	Plant height	Aboveground weight	Understan d weight	Total weight	Nodule number
NNS	V2	2.741	39.347	1.393	34.010	14.460	1.762	1.118	2.880	34
NNS	R2	2.518	34.587	1.642	34.335	27.360	3.138	1.266	4.404	51
NNS	R4	3.041	36.857	1.528	35.952	30.440	5.148	1.423	6.571	46
NNS	R8	/	/	1.251	36.322	30.960	/	1.517	/	/
SNS	V2	3.160	39.990	2.790	37.768	13.240	1.383	1.410	2.793	817
SNS	R2	2.096	39.941	2.270	38.157	24.420	2.459	1.610	4.069	811
SNS	R4	2.153	34.803	2.568	36.349	36.080	8.254	2.268	10.522	672
SNS	R8	/	/	0.696	36.046	38.580	/	2.656	/	/

SNS, super-nodulating soybean; *NNS*, normal nodulating soybean; *V2*, second trifoliolate stage; *R2*, full bloom stage; *R4*, full pod stage; *R8*, full maturity.

Table S2 Reads per sample obtained from 16S rRNA amplicon sequencing.

Sample ID	Compartment	Cultivar	Plant development stage	Input	Filtered	Denoised	Merged	Non-chimeric	Non-singleton
SNS1_1_S	Rhizosphere	SNS	V2	95384	88870	85061	60165	43301	42794
SNS1_2_S	Rhizosphere	SNS	V2	79996	73484	70552	52694	35370	35001
SNS1_3_S	Rhizosphere	SNS	V2	83371	76655	74375	60035	44403	44169
SNS1_4_S	Rhizosphere	SNS	V2	81879	75859	71015	36584	32279	31757
SNS1_5_S	Rhizosphere	SNS	V2	85764	79222	75163	47831	42969	42561
NNS1_1_S	Rhizosphere	NNS	V2	87375	81251	77299	48036	37068	36602
NNS1_2_S	Rhizosphere	NNS	V2	83860	77335	72880	39508	33172	32716
NNS1_3_S	Rhizosphere	NNS	V2	84532	77912	74195	45898	37597	37200
NNS1_4_S	Rhizosphere	NNS	V2	73206	67436	63470	33308	28628	28296
NNS1_5_S	Rhizosphere	NNS	V2	83743	77134	72483	36357	32239	31746
CK1_1_S	Bulk soil	\	V2	86405	79626	74413	35162	31837	31151
CK1_2_S	Bulk soil	\	V2	87560	80645	75822	36995	31596	30835
CK1_3_S	Bulk soil	\	V2	97312	89952	84620	41915	36679	35879
SNS1_1_N	Nodule	SNS	V2	65894	60570	60317	60007	59964	59963
SNS1_2_N	Nodule	SNS	V2	67376	62029	61831	61425	61354	61350
SNS1_3_N	Nodule	SNS	V2	68026	62755	62556	62166	61738	61736
NNS1_1_N	Nodule	NNS	V2	103168	95023	94896	94760	94759	94759
NNS1_2_N	Nodule	NNS	V2	62229	57816	57693	57546	57525	57525
NNS1_3_N	Nodule	NNS	V2	60189	55649	55540	55389	55386	55386
SNS1_1_R	Root	SNS	V2	65470	59942	59078	54848	53075	53031
SNS1_2_R	Root	SNS	V2	75386	69568	68047	30981	25314	25090
SNS1_3_R	Root	SNS	V2	60817	56090	55437	51375	49762	49745
NNS1_1_R	Root	NNS	V2	73154	67551	64586	35381	28513	28134
NNS1_2_R	Root	NNS	V2	79915	73949	69226	35289	29756	29306
NNS1_3_R	Root	NNS	V2	89301	82472	77951	43462	33493	32767

SNS2_1_S	Rhizosphere	SNS	R2	110408	96908	90629	49327	41643	40697
SNS2_2_S	Rhizosphere	SNS	R2	131734	113474	108362	78435	68397	67770
SNS2_3_S	Rhizosphere	SNS	R2	128768	110467	107202	87847	74450	74142
SNS2_4_S	Rhizosphere	SNS	R2	130184	113434	108534	73633	65391	64546
NNS2_1_S	Rhizosphere	NNS	R2	131236	113754	109684	86603	68470	68086
NNS2_2_S	Rhizosphere	NNS	R2	134676	117798	111581	69738	57021	56254
NNS2_3_S	Rhizosphere	NNS	R2	136840	119222	114549	84398	65144	64630
NNS2_4_S	Rhizosphere	NNS	R2	141943	123486	114799	58774	51919	50624
CK2_1_S	Bulk soil	\	R2	125972	110178	102721	47450	41410	40216
CK2_2_S	Bulk soil	\	R2	135403	117744	109505	51554	45189	43830
CK2_3_S	Bulk soil	\	R2	129241	112613	104542	46661	41267	39996
SNS2_1_R	Root	SNS	R2	123325	108674	107106	87577	80187	80107
SNS2_2_R	Root	SNS	R2	130625	114072	111942	73285	57123	56873
SNS2_3_R	Root	SNS	R2	114916	100392	99326	90695	86581	86553
NNS2_1_R	Root	NNS	R2	133242	116366	112847	62768	47288	46940
NNS2_2_R	Root	NNS	R2	130206	113010	109715	72204	51463	51158
NNS2_3_R	Root	NNS	R2	131016	113933	111341	58916	50071	49890
SNS2_1_N	Nodule	SNS	R2	92690	80666	80014	78286	77429	77414
SNS2_2_N	Nodule	SNS	R2	121548	106133	105700	104887	104872	104871
SNS2_3_N	Nodule	SNS	R2	119555	104547	104048	103248	103230	103229
NNS2_1_N	Nodule	NNS	R2	100323	87814	87270	86312	86254	86254
NNS2_2_N	Nodule	NNS	R2	123160	107550	107056	106289	106259	106258
NNS2_3_N	Nodule	NNS	R2	106705	93330	92790	91801	91739	91738
SNS3_1_S	Rhizosphere	SNS	R4	141760	132001	124937	84357	69145	67931
SNS3_2_S	Rhizosphere	SNS	R4	144378	134429	129135	100339	72175	71358
SNS3_3_S	Rhizosphere	SNS	R4	143253	133756	127383	89753	66045	65159
NNS3_1_S	Rhizosphere	NNS	R4	137409	127833	119768	62286	51607	50346
NNS3_2_S	Rhizosphere	NNS	R4	142382	131436	124042	71827	53251	51909
NNS3_3_S	Rhizosphere	NNS	R4	138269	128399	119880	59254	48339	46788

CK3_1_S	Bulk soil	\	R4	135176	126463	117475	53759	48132	46750
CK3_2_S	Bulk soil	\	R4	134292	124384	116404	59135	49346	47864
CK3_3_S	Bulk soil	\	R4	145170	134418	125917	62741	55648	54235
SNS3_1_R	Root	SNS	R4	134602	124463	119843	75034	51828	51054
SNS3_2_R	Root	SNS	R4	143664	132940	128549	79250	53090	52368
SNS3_3_R	Root	SNS	R4	140697	129846	125868	94987	64796	63961
NNS3_1_R	Root	NNS	R4	144639	134056	128297	76033	50202	49052
NNS3_2_R	Root	NNS	R4	136651	126318	123783	106469	95617	95388
NNS3_3_R	Root	NNS	R4	140957	130486	126901	104622	90778	90460
SNS3_1_N	Nodule	SNS	R4	134885	124687	123953	122880	119441	119426
SNS3_2_N	Nodule	SNS	R4	148199	137135	136505	135390	134221	134209
SNS3_3_N	Nodule	SNS	R4	144955	134753	133930	132331	131332	131325
NNS3_1_N	Nodule	NNS	R4	131277	121497	121080	120356	119892	119886
NNS3_2_N	Nodule	NNS	R4	136670	126081	125702	125286	124894	124892
NNS3_3_N	Nodule	NNS	R4	139961	129276	128686	127850	127309	127300
SNS4_1_S	Rhizosphere	SNS	R8	125860	120984	117653	104292	94155	93918
SNS4_2_S	Rhizosphere	SNS	R8	134065	129384	120873	82393	70859	69244
SNS4_3_S	Rhizosphere	SNS	R8	128696	123187	115370	81194	70537	69373
NNS4_1_S	Rhizosphere	NNS	R8	132332	126029	117699	80517	67644	66645
NNS4_2_S	Rhizosphere	NNS	R8	128752	123739	114197	69370	58615	57091
NNS4_3_S	Rhizosphere	NNS	R8	135221	129930	121993	85997	71673	70648
CK4_1_S	Bulk soil	\	R8	148151	142548	130259	74946	65810	63693
CK4_2_S	Bulk soil	\	R8	120007	115261	111958	99203	94565	94319
CK4_3_S	Bulk soil	\	R8	129941	125148	114600	67040	59618	57969
SNS4_1_R	Root	SNS	R8	128007	122909	119755	107815	87716	87545
SNS4_2_R	Root	SNS	R8	127906	122976	120047	109835	93559	93400
SNS4_3_R	Root	SNS	R8	140077	134401	130246	114382	83680	83276
NNS4_1_R	Root	NNS	R8	126443	122013	117261	88598	60933	60313
NNS4_2_R	Root	NNS	R8	125815	120839	115928	89856	58499	57977

NNS4_3_R	Root	NNS	R8	125855	120725	117768	105127	84196	84020
SNS4_1_N	Nodule	SNS	R8	99638	95822	94853	93594	92736	92722
SNS4_2_N	Nodule	SNS	R8	105427	101337	99889	96704	89463	89413
SNS4_3_N	Nodule	SNS	R8	99829	96037	94830	92711	88660	88639
NNS4_1_N	Nodule	NNS	R8	98308	94424	92939	89963	86991	86955
NNS4_2_N	Nodule	NNS	R8	110664	106028	103951	97857	83641	83500
NNS4_3_N	Nodule	NNS	R8	99383	95477	93788	89838	78672	78585

SNS, super-nodulating soybean; *NNS*, normal nodulating soybean; *V2*, second trifoliolate stage; *R2*, full bloom stage; *R4*, full pod stage; *R8*, full maturity.

Table S3 The α-diversity of bacterial communities inhabiting bulk soil, rhizosphere compartment, root endosphere compartment, and nodule endosphere compartment (means ± SD**).** Different letters in the same column indicate significant differences at $P < 0.05$ between treatments at a single root-associated compartment according to *Duncan's test*.

No.	**Compartment**	**Cultivar**	**Plants developmental stages**	Chao1
1	bulk soil (n = 12)	/	V2 (n = 3)	3666.03 ± 331.34a
2		/	R2 (n = 3)	5021.60 ± 179.08a
3		/	R4 (n = 3)	5429.32 ± 64.91a
4		/	R8 (n = 3)	4536.32 ± 765.18a
5	Rhizosphere (n = 30)	SNS	V2 (n = 5)	2508.82 ± 503.04d
6		NNS	V2 (n = 5)	2854.17 ± 199.33cd
7		SNS	R2 (n = 4)	3492.29 ± 621.83bcd
8		NNS	R2 (n = 4)	3867.05 ± 868.77bc
9		SNS	R4 (n = 3)	4377.55 ± 487.97ab
10		NNS	R4 (n = 3)	5177.50 ± 100.81a
11		SNS	R8 (n = 3)	4077.05 ± 1284.16abc
12		NNS	R8 (n = 3)	4425.49 ± 343.18ab
13	Root (n = 24)	SNS	V2 (n = 3)	925.81 ± 393.55d
14		NNS	V2 (n = 3)	2238.69 ± 237.93abc
15		SNS	R2 (n = 3)	1131.41 ± 428.41cd
16		NNS	R2 (n = 3)	2039.01 ± 220.44bcd
17		SNS	R4 (n = 3)	3348.97 ± 183.83a
18		NNS	R4 (n = 3)	2862.28 ± 1000.64abc
19		SNS	R8 (n = 3)	1788.31 ± 379.27bcd
20		NNS	R8 (n = 3)	2241.78 ± 582.86abc
21	Nodule (n = 24)	SNS	V2 (n = 3)	120.82 ± 4.63cd
22		NNS	V2 (n = 3)	26.17 ± 1.31d

23	SNS	R2<br>(n = 3)	149.62 ± 15.44cd
24	NNS	R2<br>(n = 3)	99.14 ± 35.60d
25	SNS	R4<br>(n = 3)	381.12 ± 39.19bc
26	NNS	R4<br>(n = 3)	221.21 ± 53.96cd
27	SNS	R8<br>(n = 3)	567.00 ± 176.93b
28	NNS	R8<br>(n = 3)	1007.94 ± 255.58a
Compartment		F = 240.141	P < 0.01
Cultivar		F = 6.849	P = 0.01
Plants developmental stages		F = 18.440	P < 0.01
Compartment × Cultivar		F = 1.370	P = 0.263
Compartment × Stages		F = 4.429	P = 0.01
Cultivar × Stages		F = 0.580	P = 0.631
Compartment × Cultivar × Stages		F = 1.355	P = 0.250

Results (P values) of repeated measures ANOVA are shown below.

SNS, super-nodulating soybean; *NNS*, normal nodulating soybean; *V2*, second trifoliolate stage; *R2*, full bloom stage; *R4*, full pod stage; *R8*, full maturity.

Table S4 Permutational multivariate analysis of variance (PERMANOVA) using distance matrices.

Variables	R2		*P*	
	Bray	Jaccard	Bray	Jaccard
Stage	0.09224	0.07521	0.007	0.001
V2-vs-R2	0.032225	0.039869	0.218	0.033
V2-vs-R4	0.048113	0.060685	0.082	0.006
V2-vs-R8	0.089915	0.087763	0.004	0.001
R2-vs-R4	0.055607	0.058515	0.057	0.004
R2-vs-R8	0.106928	0.095756	0.004	0.001
R4-vs-R8	0.057119	0.067227	0.074	0.002
Microhabitat	0.42986	0.12926	0.001	0.001
Bulk_soil-vs-Rhizosphere	0.13558	0.079469	0.001	0.001
Bulk_soil-vs-Root	0.281309	0.168817	0.001	0.001
Bulk_soil-vs-Nodule	0.673703	0.197598	0.001	0.001
Rhizosphere-vs-Root	0.182857	0.117107	0.001	0.001
Rhizosphere-vs-Nodule	0.476856	0.171123	0.001	0.001
Root-vs-Nodule	0.322756	0.120403	0.001	0.001
Cultivar	0.16889	0.01584	0. 078	0.048

V2, second trifoliolate stage; *R2*, full bloom stage; *R4*, full pod stage; *R8*, full maturity. *P* is the significance level.

Table S5 microbial community variance explained by nodule number and plant age according to Canonical analysis of principal coordinates (CAP) using the Jaccard metric.

Variables included			R_{adj}^2	anova.*P*
ASV	Rhizosphere	Nodule number	0.02739	0.040
		Plant age	0.05435	0.003
	Root	Nodule number	0.07158	0.025
		Plant age	0.0436	0.081
	Nodule	Nodule number	*value<0	0.535
		Plant age	0.058	0.089
Genus	Rhizosphere	Nodule number	0.04282	0.062
		Plant age	0.09126	0.008
	Root	Nodule number	0.12307	0.040
		Plant age	0.01457	0.345
	Nodule	Nodule number	*value<0	0.568
		Plant age	0.03031	0.220

Table S6 Correlations between bacterial communities and nodule number in root-associated microhabitats.

	Variables included	Mantel statistic r	P
Rhizosphere	Nodule number	0.1344	0.034
	Plant age	0.4588	<0.01
Root	Nodule number	0.1766	0.040
	Plant age	0.08441	0.135
Nodule	Nodule number	*value<0	0.848
	Plant age	*value<0	0.513

The Mantel statistic based on Spearman's rank correlation method. Variables were calculated based on the Euclidean distance, and the microbial community structures (ASV level) were calculated based on the Jaccard distance. *P* is the significance level.

References

1Li Y, Pei Y, Shen Y, Zhang R, Kang M, Ma Y et al. Progress in the Self-Regulation System in Legume Nodule Development-AON (Autoregulation of Nodulation). International Journal of Molecular Sciences2022.

2Wang Z, Wang L, Wang Y, Li X. The NMN Module Conducts Nodule Number Orchestra. iScience. 2020;23(2):100825. 'doi:'10.1016/j.isci.2020.100825.

3Searle IR, Men AE, Laniya TS, Buzas DM, Iturbe-Ormaetxe I, Carroll BJ et al. Long-distance signaling in nodulation directed by a CLAVATA1-like receptor kinase. SCIENCE. 2003;299(5603):109-12. 'doi:'10.1126/science.1077937.

4Kim MY, Van K, Lestari P, Moon JK, Lee SH. SNP identification and SNAP marker development for a GmNARK gene controlling supernodulation in soybean. THEOR APPL GENET. 2005;110(6):1003-10. 'doi:'10.1007/s00122-004-1887-2.

5Arai MCUM, Hayashi M, Takahashi M, Shimada S, Harada K. Expression and Sequence Analysis of Systemic Regulation Gene for Symbiosis, Nts1/Gmnark in Supernodulating Soybean Cultivar, Sakukei 4. BREEDING SCI. 2005;2(55):147-52

6Takahashi M, Nakayama N, Arihara J. Plant Nitrogen Levels and Photosynthesis in the Supernodulating (Glycine Max L. Merr.) Cultivar ‘Sakukei 4’. PLANT PROD SCI. 2005;4(8):412-8

7Dombrowski N, Schlaeppi K, Agler MT, Hacquard S, Kemen E, Garrido-Oter R et al. Root microbiota dynamics of perennial Arabis alpina are dependent on soil residence time but independent of flowering time. The ISME Journal. 2017;11(1):43-55. 'doi:'10.1038/ismej.2016.109.

8Edwards JA, Santos-Medellín CM, Liechty ZS, Nguyen B, Lurie E, Eason S et al. Compositional

shifts in root-associated bacterial and archaeal microbiota track the plant life cycle in field-grown rice. PLOS BIOL. 2018;16(2):e2003862. 'doi:'10.1371/journal.pbio.2003862.

9Zhao M, Zhao J, Yuan J, Hale L, Wen T, Huang Q et al. Root exudates drive soil-microbe-nutrient feedbacks in response to plant growth. Plant, Cell & Environment. 2021;44(2):613-28. 'doi:'https://doi.org/10.1111/pce.13928.
